# T‐Cell Remodeling in Renal Fibrosis: From Acute Injury to Chronic Kidney Disease

**DOI:** 10.1002/advs.78008

**Published:** 2026-09-27

**Authors:** Qianhui Li, Junting Guan, Yifan Song, Hongxia Yang, Guangtao Li, Xiaodong Zhao, Jiaxin Liu, Bin Liu, Yang Tan, Honglan Zhou, Yang‐He Zhang, Yishu Wang

**Affiliations:** ^1^ Key Laboratory of Pathobiology Ministry of Education College of Basic Medical Sciences Jilin University Changchun China; ^2^ Department of Urology The First Hospital of Jilin University Changchun China; ^3^ Department of Colorectal Tumor Surgery The Second Affiliated Hospital of Harbin Medical University Harbin China

**Keywords:** biomarkers, chronic kidney disease, immune remodeling, renal fibrosis, T‐cell exhaustion, T lymphocytes

## Abstract

Renal fibrosis is a final common pathway in chronic kidney disease (CKD), yet available therapies rarely reverse established scarring. T lymphocytes are now recognized as dynamic regulators of this process rather than simple inflammatory bystanders. This Review integrates evidence from experimental kidney injury models, human biopsy studies, single‐cell RNA sequencing, spatial transcriptomics, and biomarker analyses to establish a three‐stage framework for T‐cell remodeling during the transition from acute kidney injury (AKI) to CKD. In Stage 1, chemokine gradients recruit T cells into injured kidney tissue, where effector activation, cytotoxic injury, paracrine inflammation, and metabolic adaptation shape early outcomes. In Stage 2, unresolved injury favors immune imbalance, including T helper 17/regulatory T cell disequilibrium, dendritic cell–T cell crosstalk, and tertiary lymphoid structure formation. In Stage 3, exhausted and tissue‐resident memory T cells coexist with cellular senescence, mitochondrial stress, and profibrotic signaling. Across these stages, recurrent pathways, including PI3K/Akt/mTORC1, TGF‐β/Smad3, IFN‐γ/STAT1, and IL‐15/CD122, connect immune remodeling to structural fibrosis in a manner that depends on timing, cell type, and tissue context. This framework may help refine staging based on biomarkers and guide the development of therapies matched to the dominant immune program.

## Graphical abstract

This Review presents renal fibrosis as a sequence of overlapping T‐cell programs, from chemokine‐guided recruitment and metabolic activation to maladaptive polarization, tertiary lymphoid structure formation, tissue residency, exhaustion, and senescence. Human kidney and experimental evidence identify candidate biomarkers and therapeutic entry points, clarifying how T cells shape the transition from acute kidney injury to chronic kidney disease.

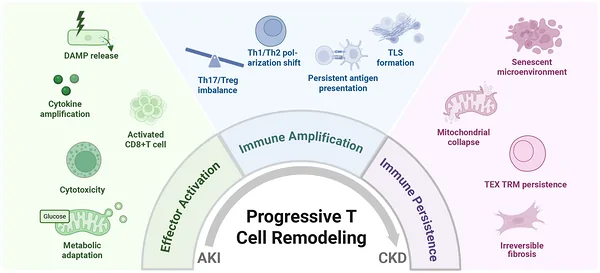

## 1 Introduction

Chronic kidney disease (CKD) is a major and growing noncommunicable disease worldwide. In the Global Burden of Disease 2017 analysis, CKD affected an estimated 9.1% of the global population and imposed a substantial burden through end‐stage kidney disease, cardiovascular complications, and premature mortality [1]. Whatever the initiating insult, including diabetic kidney disease, hypertensive nephrosclerosis, immune‐mediated glomerulonephritis, ischemia, toxic injury, or maladaptive repair after acute kidney injury (AKI), progressive CKD often converges on renal fibrosis. This process is marked by tubular atrophy, interstitial inflammation, myofibroblast activation, excessive extracellular matrix deposition, vascular rarefaction, and irreversible loss of functional parenchyma [1, 2, 3, 4]. Although transforming growth factor‐β (TGF‐β)/Smad signaling and myofibroblast biology have long been central to models of renal fibrogenesis, current therapies remain largely supportive and seldom prevent or reverse established fibrosis [2, 3, 4]. This therapeutic gap argues for viewing renal fibrosis not merely as a terminal scar, but as a dynamic multicellular disease shaped by sustained inflammatory circuits, immune‐stromal crosstalk, and maladaptive repair [5].

Among immune cells, T lymphocytes are especially important because they integrate antigenic, metabolic, and cytokine cues and translate them into effector, regulatory, exhausted, or tissue‐resident states. Experimental studies show that CD4^+^ T cells contribute to fibrosis after ureteral obstruction, and that depletion or modulation of T cell subsets can alter renal inflammation and matrix remodeling [6, 7]. Regulatory T cells (Tregs) can limit kidney inflammation and fibrosis, whereas T helper 17 (Th17) cells, T helper 2 (Th2) cells, cytotoxic CD8^+^ T cells, γδ T cells, and exhausted T cells may promote or reshape disease depending on timing and local tissue cues [7, 8, 9, 10, 11, 12]. These observations argue against a single linear role for T cells in renal fibrosis. Instead, T‐cell function evolves over time, moving from early inflammatory amplification to immune dysregulation, tissue residency, exhaustion, and chronic profibrotic signaling within a broader kidney microenvironment in which immune‐cell networks [13], lymphocyte‐innate cell interactions [14], and epithelial, endothelial, and stromal compartments jointly shape injury, repair, and fibrotic progression [15].

Recent single‐cell RNA sequencing (scRNA‐seq) and spatial transcriptomic studies have sharpened this view by showing that renal immune states vary across disease stages and anatomical niches [16, 17, 18, 19, 20]. These data support a staged immune framework, while also underscoring the need to validate trajectories inferred from animal models in longitudinal human samples.

T cell involvement in renal fibrosis is therefore best considered as a temporal process. Soon after injury, chemokine gradients recruit circulating T cells into the kidney. Effector CD4^+^ and CD8^+^ T‐cell programs can then promote kidney parenchymal injury through IFN‐γ, TNF‐α, granzyme B, perforin, and related inflammatory mediators [6, 7, 21, 22]. During the subacute phase, continuing antigen exposure and metabolic stress disturb immune balance, particularly through Th17/Treg disequilibrium [8, 12], crosstalk between dendritic cells (DCs) and T cells [21, 23], and the progressive organization of tertiary lymphoid structures (TLS) [24]. TLS are ectopic immune niches that sustain local antigen presentation, T–B cell cooperation, cytokine production, and chronic inflammation in nonlymphoid organs [25, 26]. In kidney disease, TLS formation has been linked to progressive tissue damage and is regulated in part by IL‐17A signaling [24]. At later stages, chronic antigenic and metabolic stimulation can drive exhausted T‐cell (TEX) phenotypes in kidney failure and transplantation [10, 11], alongside tissue‐resident memory T‐cell (TRM) programs described in autoimmune, glomerular, and fibrotic kidney settings [27, 28, 29]. In some transplant contexts, TEX phenotypes are associated with improved allograft outcomes, suggesting that exhaustion may restrain acute immunopathology while still permitting chronic inflammatory persistence [10].

Despite this growing evidence, many studies and reviews still focus on individual T cell subsets rather than on how T‐cell programs change as kidney injury progresses. A staged framework is useful because it brings recruitment, activation, immune imbalance, exhaustion, and tissue residency into a single mechanistic narrative.

The main novelty of this Review is a stage‐resolved framework of T‐cell remodeling in renal fibrosis that integrates established kidney‐immune and TLS concepts with recent single‐cell and spatial evidence. This framework links T‐cell recruitment, antigen‐presenting cell (APC)‐driven polarization, TLS maturation, metabolic rewiring, exhaustion, senescence, TRM persistence, biomarker readouts, and therapeutic timing across the progression from AKI to CKD. The three stages represent dominant or transitional functional windows rather than fixed chronological or clinical boundaries, because chemokine and retention pathways can bridge more than one disease stage.

Human kidney atlas studies provide direct patient‐tissue support for this framework. The Kidney Precision Medicine Project (KPMP) atlas maps healthy and injured kidney cell states and niches [19], multi‐omic and spatial profiling links CKD progression to fibrotic microenvironments [18], and the human kidney fibrosis atlas identifies myofibroblast‐lineage programs relevant to T‐cell–stromal crosstalk [20]. Taken together, these human and experimental data support a three‐stage model designed to organize heterogeneous evidence, identify testable biomarkers, and clarify when specific interventions may be biologically plausible, rather than to impose a rigid clinical classification.

## 2 Overview of the Three‐Stage Mechanistic Framework

Renal fibrosis is better viewed as a sequence of overlapping immune states than as a set of fixed time windows. In this Review, the three stages are defined by the dominant T‐cell program, renal niche, candidate readouts, and therapeutic vulnerabilities, as summarized in Table 1 and illustrated in Figure 1. Stage 1 centers on T‐cell recruitment and effector activation during AKI‐to‐CKD transition [22], with metabolic adaptation considered an accompanying effector program [30], whereas Stage 2 reflects unresolved inflammation, characterized by Th17/Treg imbalance [31], APC–T‐cell crosstalk [23], and TLS formation [32]. Stage 3 is marked by TEX states in kidney failure or transplantation [10, 11, 33], TRM persistence in renal immune injury [27], and senescence‐associated remodeling [34].

**TABLE 1:** T cell states and supporting evidence across the three stages of renal fibrosis.

Stage	Key features	Representative pathways	Evidence
Stage 1: Effector activation	Recruitment, cytotoxicity, and metabolic adaptation, representing the early immune activation phase	PI3K/Akt/mTORC1; HIF‐1α; Ca^2^^+^–NFAT; NF‐κB	**Human**: Biopsy, single‐cell, spatial, and cross‐tissue data support T‐cell infiltration, immune niches, and GZMK^+^ inflammatory CD8^+^ states. **Mouse kidney**: AKI, IRI, and UUO models define the kinetics of recruitment, cytotoxicity, and metabolic signaling. **Gap**: Rapid kinetics and high‐GzmB cytotoxicity remain model‐dominant.
Stage 2: Immune dysregulation	Th17/Treg imbalance, APC–T‐cell crosstalk, and TLS formation, contributing to a chronic inflammatory niche	SGK1/RORγt; Orai1/NFAT; mTOR/Foxp3; NLRP3	**Human**: Cohort, biopsy, and spatial‐omic studies support Th17/Treg imbalance, APC–T‐cell niches, and TLS in selected kidney diseases. **Mouse kidney**: UUO, IRI, cisplatin, and high‐salt models define TLS timing and support causal roles for SGK1, Orai1/NFAT, and NLRP3. **Gap**: Temporal ordering and pathway‐specific intervention evidence remain largely preclinical.
Stage 3: Exhaustion and tissue persistence	Exhaustion, tissue residency, senescence, and metabolic failure, associatedwith persistent inflammation and fibrosis	TOX; IL‐15/CD122; CXCR6/CXCL16; IFN‐γ/STAT1	**Human**: Transplant, kidney‐failure, and autoimmune datasets support TEX/TRM accumulation, checkpoint remodeling, and senescence‐associated states. **Mouse/cross‐organ**: IL‐15/CD122, CXCR6/CXCL16, NLRP3–TRM, and TOX trajectories provide causal or conceptual support. **Gap**: The full TEX trajectory lacks longitudinal validation in common human CKD.

Note: Evidence scope indicates whether support comes from human kidney tissue/cohorts, mouse renal injury models, or cross‐organ mechanisms; it is not a formal evidence‐quality grading. Abbreviations: AKI, acute kidney injury; APC, antigen‐presenting cell; CKD, chronic kidney disease; IRI, ischemia–reperfusion injury; TEX, exhausted T cell; TLS, tertiary lymphoid structure; TRM, tissue‐resident memory T cell; Treg, regulatory T cell; UUO, unilateral ureteral obstruction.

**FIGURE 1:**
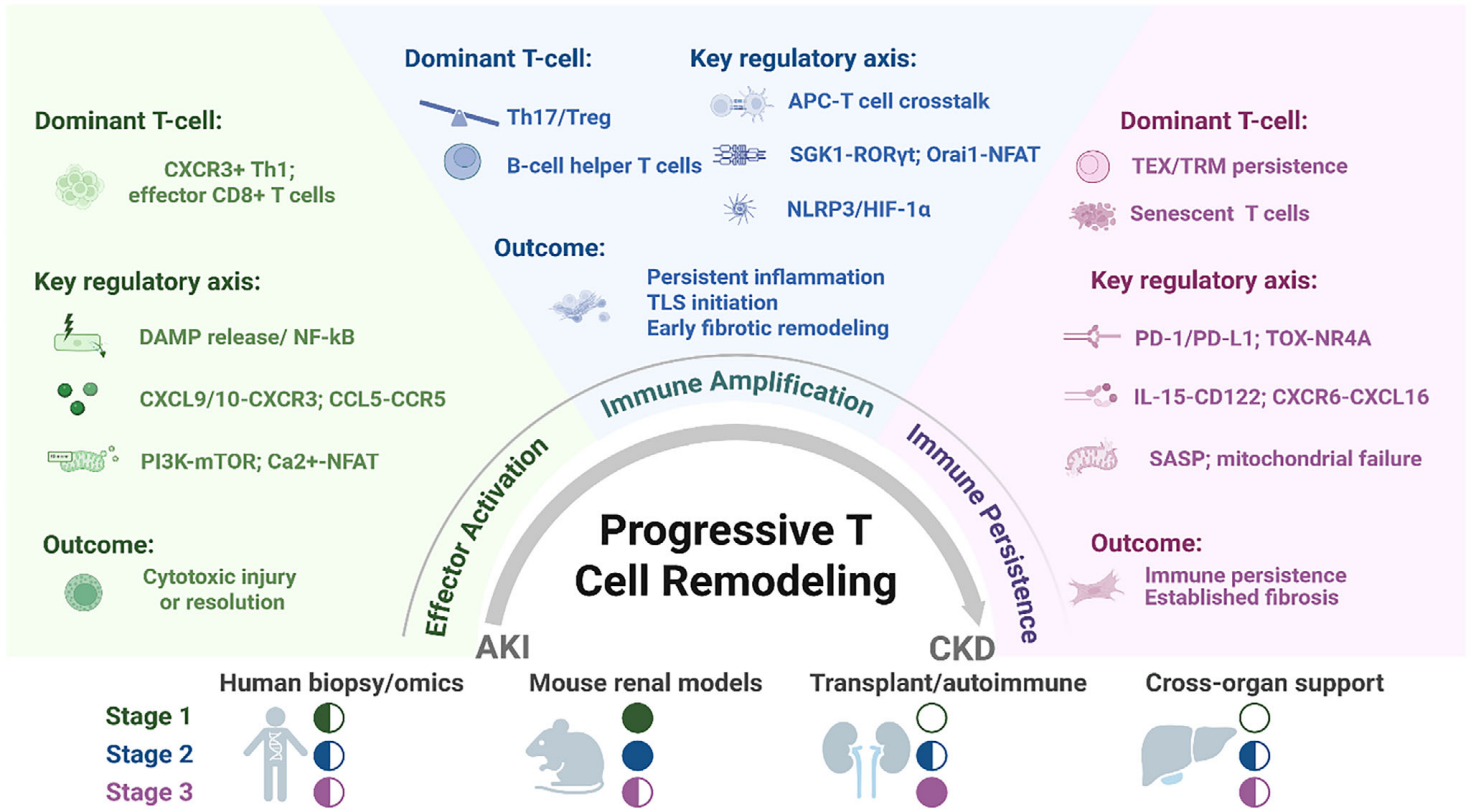
T‐cell remodeling during progression from AKI to CKD. Renal fibrosis evolves through overlapping immune programs rather than discrete linear phases. Early injury recruits and activates effector T cells; unresolved inflammation promotes Th17/Treg imbalance, APC–T‐cell crosstalk, and TLS formation; and advanced disease is marked by TEX/TRM persistence, senescence‐associated remodeling, and mitochondrial stress. The lower evidence‐scope summary indicates the relative support from human biopsy/omics, mouse renal models, transplant/autoimmune studies, and cross‐organ mechanisms. Abbreviations: AKI, acute kidney injury; APC, antigen‐presenting cell; CKD, chronic kidney disease; TEX, exhausted T cell; TLS, tertiary lymphoid structure; TRM, tissue‐resident memory T cell; Treg, regulatory T cell.

Human atlas studies provide patient‐tissue anchors for this model [19, 20], but stagetiming and boundaries are expected to differ by disease etiology, sampling time, and treatment exposure [20]. Because rodent unilateral ureteral obstruction (UUO), ischemia–reperfusion injury (IRI), and cisplatin models define causal timing over days to weeks, whereas human CKD usually evolves over years, Table 1 also indicates the broad evidence scope for each stage [35, 36]. Thus, the framework is intended as a mechanistic and translational map rather than a replacement for existing clinical CKD staging.

## 3 Stage 1: Early Effector Programming and Fibrotic Commitment

The initiation of renal fibrosis involves immune programming as well as structural injury. After ischemic, toxic, metabolic, or immune‐mediated insults, injured renal tissue creates chemokine and cytokine gradients that draw circulating T cells into the kidney. Metabolic adaptation, cytotoxic activation, and inflammatory commitment in these cells then influence whether injury resolves or progresses [22, 30]. The chemokine axes that shape this early entry program are organized in Table 2. Human atlas data provide complementary support for injury‐associated epithelial and immune niches in patient tissue [19].

**TABLE 2:** Chemokine axes guiding T‐cell recruitment, regulation, and tissue retention in renal fibrosis.

Chemokine axis	Associated T cell	Renal source or trigger	Functional role in fibrosis	Stage	Refs
CXCL9/CXCL10/CXCL11–CXCR3	Th1, effector CD8^+^ T cells, CXCR3^+^ Tregs	IFN‐γ/NF‐κB‐activated proximal TECs	Promotes recruitment of inflammatory T cells; CXCL10 may also limit fibroblast activation	Stage 1	[37, 38, 39]
CCL5–CCR5	Cytotoxic CD8^+^ T cells	NET‐licensed macrophages	Sustains feed‐forward immune‐cell recruitment	Stage 1–2	[40]
CCL20–CCR6	Th17 cells	Inflamed TECs, stromal cells	Supports Th17 trafficking and IL‐17‐associated fibrosis	Stage 2	[41]
CCL28–CCR10; CXCR3‐associated	Tregs	ALKBH5‐regulated TECs	Promotes regulatory T‐cell homing and tissue repair	Stage 2	[42]
CXCL16–CXCR6	TRM and memory‐like CD8^+^ T cells	Injured TECs	Maintains tissue residency and survival	Stage 3	[28, 43]

Abbreviations: NET, neutrophil extracellular trap; TEC, tubular epithelial cell; TRM, tissue‐resident memory T cell; Treg, regulatory T cell.

### 3.1 Chemokine‐Guided T‐Cell Recruitment

Chemokine‐guided recruitment helps establish the early immune landscape of the injured kidney. In inflammatory injury and transplantation‐associated settings, CXCL9/CXCL10–CXCR3 marks recruitment of Th1 and CD8^+^ T cells [37]. However, CXCL10 can also exert antifibrotic effects on fibroblasts when preserved [38, 39]. The CCL5–CCR5 axis supports a self‐reinforcing CD8^+^ T‐cell recruitment loop in obstructive fibrosis. This process involves a relay in which neutrophil extracellular traps (NETs) stimulate macrophages to produce CCL5, thereby promoting further T‐cell recruitment [40]. CCL20–CCR6 is linked to Th17 trafficking and susceptibility to IgA nephropathy [41], whereas CXCL16–CXCR6 becomes more closely associated with tissue retention in later or chronic settings [43]. Treg recruitment is supported by CCL28–CCR10 in renal IRI models [42]. Table 2 summarizes these chemokine axes according to the responding T‐cell state, renal trigger, disease context, and dominant functional window. Although chemokines provide the main directional cues for early T‐cell entry, cytokine and APC‐derived signals further support adhesion, extravasation, local retention, and reactivation. Thus, early recruitment should be viewed as a coordinated process involving chemotaxis, inflammatory licensing, and local antigen presentation.

### 3.2 Early T‐Cell Infiltration: Dynamics and Effector Mechanisms

T cells enter the renal stroma rapidly after injury. In a UUO model, CD8^+^ T cells increase markedly within the first week and form a precursor population for early tissue infiltration [44]. scRNA‐seq studies further show that these early T cells are heterogeneous and progressively acquire effector features, with contraction of the initial population and expansion of effector and proliferating subsets [22].

Once in the kidney, infiltrating T cells can promote fibrotic remodeling through both cytotoxic and paracrine mechanisms. CD8^+^ T cells recognize stress‐associated MHC class I molecules and NKG2D ligands on podocytes, followed by release of granzyme B and perforin [45]. This cytotoxic interaction contributes to podocyte apoptosis and disruption of the glomerular filtration barrier [45]. Activated CD4^+^ and CD8^+^ T cells also produce IFN‐γ, TNF‐α, IL‐17, and IL‐6, which activate NF‐κB and MAPK signaling in resident renal cells and sustain inflammation when injury persists [21, 46].

T cell‐derived IL‐17, TNF‐α, and TGF‐β can activate canonical TGF‐β/Smad signaling and promote myofibroblast‐like remodeling of tubular epithelial cells (TECs) and interstitial fibroblasts [47, 48]. Effector memory T cells (TEMs) may remain after apparent recovery from acute injury and continue to express TNF‐α, delaying resolution and facilitating progression from AKI to CKD [49]. In clinical biopsy cohorts, tubulointerstitial T cell infiltration has been associated with subsequent estimated glomerular filtration rate (eGFR) decline, supporting its value as a prognostic tissue feature [50, 51].

Human CKD samples support parts of this model while also highlighting species differences. Renal biopsy transcriptomics show upregulation of the NKG2D ligand MICB across several forms of CKD [45]. Cross‐tissue scRNA‐seq analyses indicate that human kidney‐infiltrating CD8^+^ T cells often display a GzmK^+^/GzmB^+^/perforin^+^ phenotype dominated by cytokine production rather than the high‐GzmB cytotoxic phenotype observed in some mouse models [52]. Thus, CD8^+^ T cell injury in human CKD may depend substantially on paracrine inflammatory signaling.

### 3.3 Metabolic Adaptation during Early Effector Programming

Metabolic adaptation is a central component of early effector programming, but its contribution differs by T cell subset and disease context. Resting T cells rely mainly on mitochondrial oxidative phosphorylation (OXPHOS) for basal ATP generation. After activation, many T cells, particularly CD8^+^ effector cells and CD4^+^ naive cells, increase aerobic glycolysis [30, 53]. This shift supports ATP production, anabolic biosynthesis, and the synthesis of effector molecules such as granzyme B and perforin [30, 54].

Early infiltrating T cells are metabolically diverse. Single‐cell analyses in AKI‐to‐CKD models show heterogeneous renal T‐cell trajectories, including proliferating and effector CD8^+^ T‐cell states [22]. This observation suggests that the metabolic groundwork for chronic disease may be laid early. Clinical metabolomic studies point in the same direction: in kidney allograft recipients, urinary lactate is increased together with changes in alanine, citrate, and urea metabolism, and these profiles associate with immune‐mediated kidney injury [55].

Glycolysis is only one part of this program. Outside kidney disease, glutamine‐linked reductive carboxylation through mitochondrial IDH2 and KDM5‐dependent chromatin remodeling has been shown to shape T‐cell differentiation [56], while UGCG‐dependent glycosphingolipid synthesis supports CD8^+^ T‐cell membrane organization, TCR signaling, and cytotoxicity in tumor immunity [57]. In renal fibrosis, these pathways are best viewed as plausible metabolic extensions that may shape kidney‐infiltrating CD8^+^ T‐cell function, but they require direct kidney‐specific validation.

These metabolic adaptations sustain early effector responses and may also shape the later emergence of immune imbalance and exhaustion.

### 3.4 Key Signaling Pathways in Stage 1

Stage 1 signaling involves both inflammatory activation and metabolic adaptation, consistent with broader AKI‐to‐CKD signaling models [58]. Damage‐associated molecular patterns (DAMPs) and Toll‐like receptor (TLR) ligands, together with IL‐1β and TNF‐α, activate NF‐κB in TECs, endothelial cells, macrophages, and infiltrating T cells, inducing chemokines, adhesion molecules, IL‐6, TNF‐α, and NLRP3 priming [59, 60, 61]. Interventional evidence that IκB kinase inhibition after AKI improves renal recovery and attenuates fibrosis further supports NF‐κB as an early injury‐to‐fibrosis pathway [62].

TGF‐β/Smad signaling is also engaged during the acute‐to‐subacute phase, not only in established fibrosis. Injury‐derived TGF‐β activates Smad2/3 in TECs, podocytes, fibroblasts, and immune cells, coupling early cytokine injury to EMT‐like remodeling, myofibroblast activation, and matrix gene expression [48, 63, 64]. Together, NF‐κB and TGF‐β/Smad link early inflammation to profibrotic remodeling, while PI3K/Akt/mTORC1 and calcium‐dependent NFAT programs shape T‐cell effector and metabolic responses.

Within this inflammatory context, early T‐cell metabolic adaptation is regulated in part by PI3K/Akt/mTORC1 signaling. T‐cell receptor (TCR) and CD28 costimulation activate PI3K, recruit Akt to the plasma membrane, and relieve TSC1/2‐mediated inhibition of mTORC1 [54]. Activated mTORC1 increases glycolytic enzyme expression and cap‐dependent translation, supporting clonal expansion and effector molecule production. In renal‐fibrosis models, interventions that reduce PI3K/Akt/mTOR pathway activity have been associated with attenuation of fibrosis, although the relevant immune and renal cell types still require definition [65].

A complementary T‐cell‐intrinsic control pathway is calcium–calcineurin–NFAT signaling. TCR‐induced calcium release activates STIM1 and Orai1‐dependent store‐operated calcium entry (SOCE), which in turn activates calcineurin and promotes nuclear translocation of NFAT. NFAT signaling connects cytokine production to metabolic programming and has been implicated in Th17 polarization during progressive kidney injury [66]. NFAT5–SGK1 signaling in high‐salt conditions is related but not identical to canonical signaling through calcineurin and NFAT and is discussed in Section 4.5 [67, 68].

### 3.5 T‐Cell Interactions with the Injured Renal Microenvironment

#### 3.5.1 Direct Cytotoxicity against Podocytes

During the initiation of fibrosis, CD8^+^ T cells can attack stressed podocytes through contact‐dependent interactions, inducing apoptosis and disrupting the glomerular filtration barrier [45]. Granzyme B may also contribute to nonapoptotic remodeling by cleaving extracellular matrix components and releasing active TGF‐β1 [69]. In autoimmune glomerulonephritis, including anti‐GBM nephritis, T‐cell responses are associated with disease activity and renal inflammatory injury [60, 70].

#### 3.5.2 Tubular Epithelial Cells as Targets, Amplifiers, and Antigen‐Presenting Cells

Injured TECs are not passive targets during renal fibrosis. They upregulate MHC class II molecules, including H2‐Aa and Cd74, through JAK/STAT1 signaling, acquire antigen‐presenting capacity, and activate infiltrating T cells [49, 71]. T cell‐derived TNF‐α can disrupt TEC metabolic homeostasis, induce VCAM1 expression, and promote senescence and apoptosis. Neutrophil‐derived IL‐1β may further amplify oxidative stress through ROS‐NF‐κB signaling [49].

Tubule‐specific deletion of H2‐Ab1 reduces renal CD4^+^ T‐cell infiltration and shifts Treg/Th2 balance in UUO or folic acid‐induced renal injury, thereby attenuating fibrosis [72]. In chronic active antibody‐mediated rejection, higher intragraft granzyme B expression and circulating cytotoxic T‐cell changes correlate with proteinuria and graft injury [73]. IFN‐γ stimulates TECs to secrete CXCL10, which promotes epithelial‐mesenchymal transition (EMT) through PI3K/Akt signaling; this response can be inhibited by Cxcl10 siRNA [38]. Clinically, tubulitis severity has been linked to fibrosis progression and may outperform the i‐INT score in selected biopsy settings [74].

#### 3.5.3 Synergy with Innate Immune Cells

The effects of T cells are amplified by their interactions with other immune populations, particularly macrophages. Resident and infiltrating M1 macrophages upregulate MHC class II, promote CD4^+^ T‐cell activation, and enhance NF‐κB signaling in T cells through IL‐1 and TNF‐α. Activated T cells, in turn, release IL‐6 and TNF‐α, reinforcing NF‐κB signaling in macrophages and creating a positive feedback loop [61]. In the referenced anti‐GBM glomerulonephritis model, macrophage TLR4 loss impaired antigen presentation and was associated with an approximately 30%–40% reduction in renal T‐cell infiltration [70]. CD8^+^ T cells can also engage M2 macrophages through the CCL5–CCR1 axis, activating ATF3/FOS/EGR1 signaling and driving profibrotic polarization; this circuit can be reversed by the sodium‐glucose cotransporter 2 (SGLT2) inhibitor empagliflozin [75, 76]. Both transcriptomic and immunohistochemical analyses reveal colocalization of macrophages and T cells in injured renal tubulointerstitium, supporting local innate–adaptive immune cooperation during fibrotic progression [77, 78]. In diabetic nephropathy, CD103^+^ dendritic cells enhance CD8^+^ T‐cell activation and expansion by upregulating CD80 and CD86, whereas inhibition of dendritic cell maturation weakens this axis [79, 80]. In some settings, including ureteral obstruction, myeloid‐derived suppressor cells recruited through CCL5–CCR5 can suppress Th1 effector function by expanding Tregs, limiting Smad/Snail activation, and delaying EMT and extracellular matrix deposition [75].

Stage 1 can therefore be viewed as a phase of immune commitment, in which early recruitment, metabolic adaptation, and effector programming help determine whether renal injury resolves or advances toward chronic fibrosis. These recruitment, signaling, and metabolic programs are summarized in Figure 2.

**FIGURE 2:**
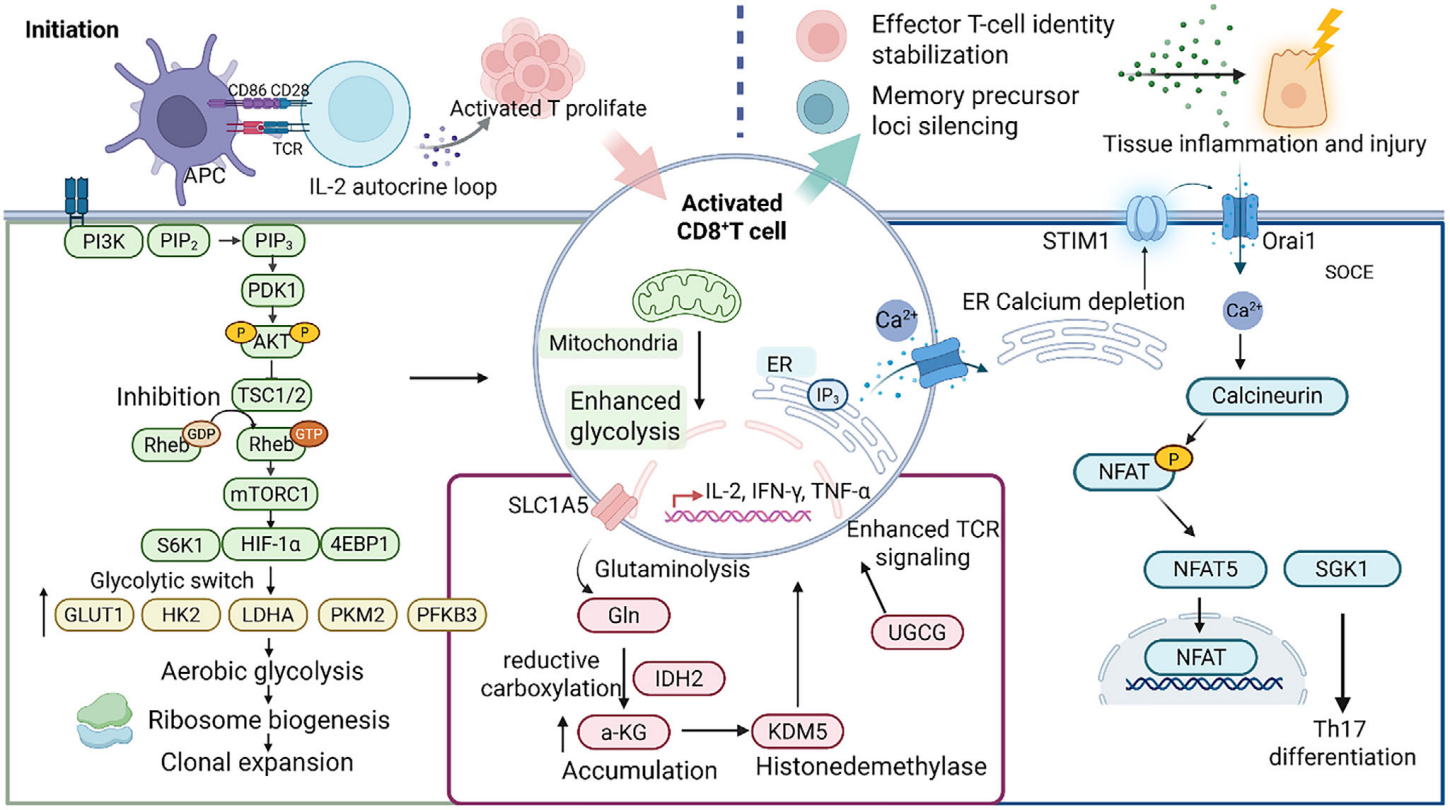
Early T‐cell activation and metabolic adaptation in the injured kidney. Antigen recognition, costimulatory signals, and chemokine gradients initiate T‐cell activation after kidney injury. PI3K/Akt/mTORC1 signaling increases glycolysis and biosynthetic activity, while glutamine metabolism and membrane lipid remodeling are shown as plausible support modules for effector differentiation and cytotoxic function. Calcium signaling through calcineurin and NFAT links TCR activation to cytokine production and may help determine whether early inflammation resolves or progresses. Abbreviations: mTORC1, mechanistic target of rapamycin complex 1; NFAT, nuclear factor of activated T cells; PI3K, phosphoinositide 3‐kinase; SOCE, store‐operated calcium entry; TCR, T cell receptor.

## 4 Stage 2: Immune Dysregulation and Amplification of Fibrosis

Stage 2 begins when early inflammation does not resolve. Antigen release from injured TECs, continued chemokine recruitment, and ongoing metabolic activation no longer support only effector expansion; instead, they promote disordered immune polarization. The major features are Th17/Treg imbalance, Th1/Th2 remodeling, DC–T cell crosstalk, and TLS formation.

### 4.1 Th17/Treg Imbalance: A Recurrent Axis of Disease Progression

During the transition from acute injury to progressive fibrosis, the balance between proinflammatory Th17 cells and anti‐inflammatory Tregs is repeatedly altered in experimental models and clinical cohorts. A shift toward Th17 predominance is therefore best regarded as a recurrent feature of progression, rather than as a universal hallmark present in every form of kidney disease.

#### 4.1.1 Th17 Cells as Profibrotic Effectors Across Models

Th17 cells are defined by RORγt expression and secretion of IL‐17A, IL‐17F, and IL‐22. In AKI‐to‐CKD, high‐salt post‐AKI, IL‐27Rα‐deficient, and obstructive kidney‐injury models, Th17/IL‐17A activation has been linked to renal inflammation, collagen deposition, tubular atrophy, and progressive fibrosis [46, 47, 66, 67]. In cisplatin‐induced renal fibrosis, IL‐17A and Foxp3 expression increased in lymphocyte aggregates at different phases, suggesting dynamic Th17/Treg involvement rather than a simple Th17‐only pattern [78]. IgA nephropathy studies further support Th17/Treg imbalance and CCR6‐linked Th17 susceptibility [41, 81], whereas anti‐GBM studies support broader T‐cell inflammatory imbalance in glomerulonephritis [60, 70].

Human data point in the same direction, although disease context should be specified. In selected human CKD cohorts, peripheral Th17/Treg imbalance, higher serum IL‐17, lower IL‐10, and endoplasmic reticulum stress markers correlate with renal dysfunction and CKD stage [82]. In patients with type 2 diabetes, serum IL‐17A levels are higher in DKD and inversely associated with eGFR [83], and model‐based evidence indicates that dapagliflozin attenuated SGK1‐linked Th17/Treg imbalance in db/db mice [84]. At the tissue level, kidney‐relevant TRM17/Th17‐like programs have been implicated in autoimmune kidney disease, where CD69^+^/RORγt^+^ TRM17 cells correlate with renal dysfunction [72]. Unlike circulating Th17 cells, renal TRM17 cells can undergo bystander activation in response to IL‐23, IL‐6, and IL‐1β without direct TCR engagement, sustaining IL‐17A release [72]. Counter‐regulatory mechanisms also exist. In folic acid‐induced AKI, galectin‐8 selectively induces apoptosis of activated Th17 cells and limits IL‐17A during progression from AKI to CKD [85].

Functionally, IL‐17A can induce inflammatory chemokine and cytokine programs in renal target cells, consistent with its broader role in renal inflammation [12, 47]. In obstructed kidneys, IL‐17A from γδ T cells and CD4^+^ Th17 cells drives CCL5/RANTES‐mediated leukocyte infiltration and renal fibrosis [46]. These effects can amplify neutrophil and macrophage infiltration and are strengthened by TNF‐α costimulation [47]. IL‐17A also promotes EMT and collagen deposition through ERK/p38 MAPK–Smad3 crosstalk [48].

#### 4.1.2 Treg Cells: Protective but Functionally Plastic

Tregs are characterized by Foxp3 expression and immunoregulatory mediators such as IL‐10 and TGF‐β. Their protective role in renal injury should be interpreted mainly as immune regulation: stable Tregs suppress effector T‐cell responses, limit inflammatory cytokine release, and support recovery or remodeling after kidney injury in Treg‐expansion and immune‐cell landscaping studies [8, 48]. Conversely, loss or depletion of Tregs is associated with greater renal inflammation and fibrosis in model‐specific studies [8, 86]. By reducing inflammatory T‐cell and macrophage activation, Tregs may indirectly restrain fibroblast activation [8, 86].

Treg stability, however, is not fixed. In the fibrotic kidney, hypoxia, metabolic stress, and STAT3 signaling stabilize HIF‐1α. HIF‐1α can activate RORγt transcription, assemble with RORγt and p300 at the IL‐17 promoter, and bind Foxp3, targeting it for proteasomal degradation [87]. Reduced SIRT3 expression in UUO datasets is associated with renal fibrosis and immune‐regulatory changes, consistent with this hypoxia‐related shift [88]. The resulting FOXP3^+^/IL‐17^+^ transitional population expresses both lineage markers but retains profibrotic properties. In UUO, histone deacetylase inhibition with trichostatin A reduces accumulation of this population and attenuates fibrosis [31]. This protective role can be lost when Treg stability declines or when TGF‐β1/Smad3 signaling becomes sustained in fibroblasts and TECs; under those conditions, TGF‐β can promote myofibroblast activation, TEC EMT, and matrix deposition rather than immune resolution [31, 48, 89].

#### 4.1.3 Th17/Treg Balance: Dynamic Regulation and Clinical Significance

The Th17/Treg ratio has emerged as a candidate indicator of fibrotic progression, although most clinical evidence remains observational. In human CKD and chronic renal failure cohorts, increased Th17/Treg ratio, higher serum IL‐17 and endoplasmic reticulum stress markers, and lower IL‐10 associate with disease severity [82, 90], while db/db diabetic kidney disease (DKD) models support SGK1‐linked Th17/Treg skewing as a modifiable inflammatory pathway [84]. In IgA nephropathy, activated Tregs correlate with better renal function, whereas tubular IL‐17A expression has been linked to more severe tubulointerstitial fibrosis [81]. These findings support further validation of Th17/Treg imbalance as a staging feature rather than a stand‐alone diagnostic test. The main molecular circuits that stabilize this imbalance are summarized in Figure 3.

**FIGURE 3:**
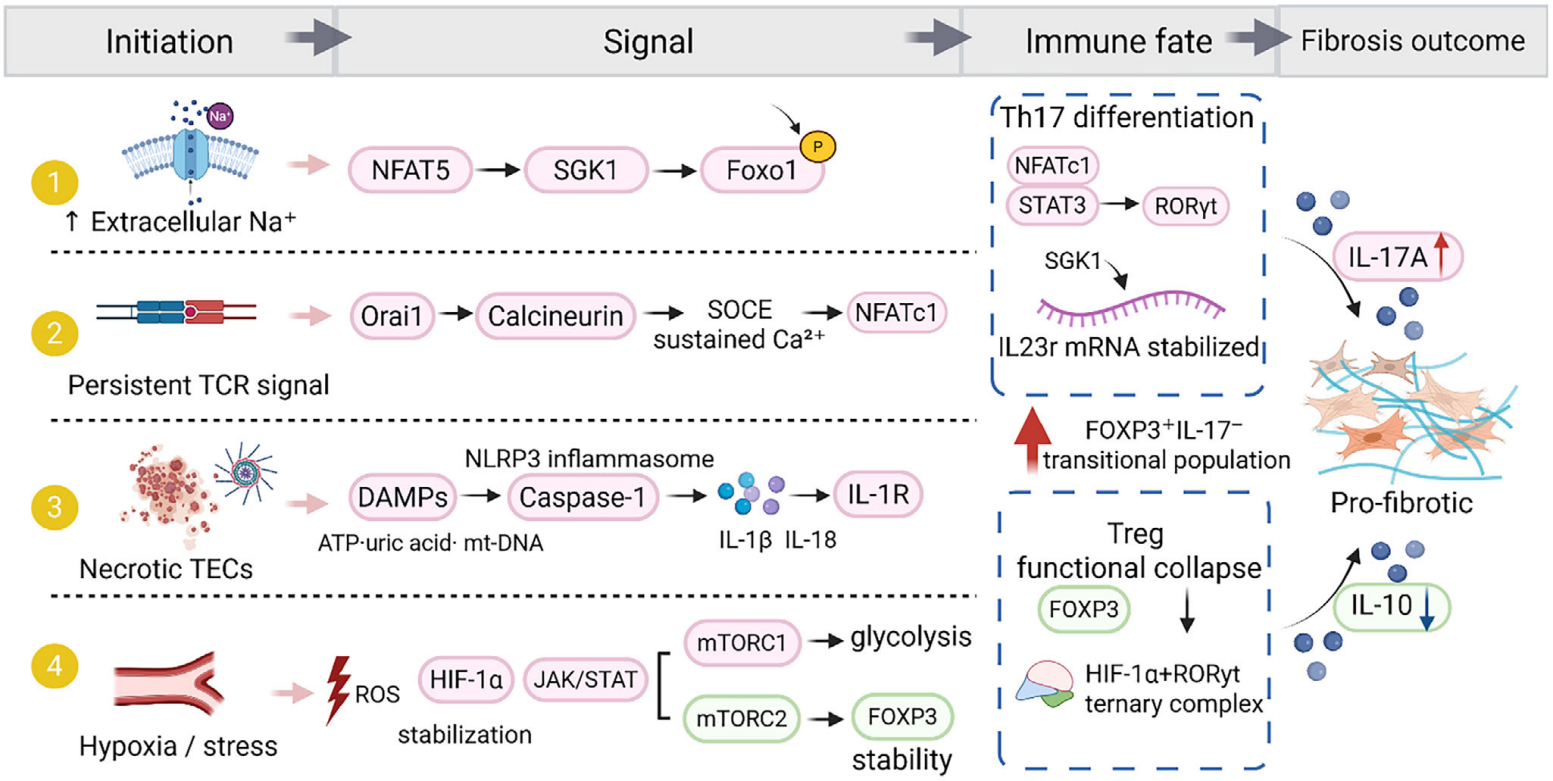
Molecular circuits associated with Th17/Treg imbalance in Stage 2 renal fibrosis. Continuing antigen stimulation, elevated extracellular sodium, damage‐associated molecular patterns, and hypoxic metabolic stress disrupt immune homeostasis. SGK1, calcium–NFAT, NLRP3 inflammasome activation, and HIF‐1α‐dependent metabolic programs favor Th17 differentiation while weakening Treg stability and suppressive function. The resulting imbalance contributes to persistent inflammation and fibrotic remodeling. Abbreviations: DAMP, damage‐associated molecular pattern; HIF‐1α, hypoxia‐inducible factor 1α; NFAT, nuclear factor of activated T cells; NLRP3, NOD‐like receptor family pyrin domain‐containing 3; SGK1, serum‐ and glucocorticoid‐regulated kinase 1.

### 4.2 Th1/Th2 Polarization and Fibrotic Context

Although the Th17/Treg axis is prominent in many studies, Th1/Th2 polarization also shapes the immune trajectory of renal fibrosis. Th1 cells are usually enriched during early inflammatory injury and can promote tubular epithelial cell apoptosis and macrophage activation [91]. By contrast, Th2 responses are context‐dependent. During acute or resolving injury, IL‐4/IL‐13 signaling may dampen type 1 inflammation and support reparative macrophage and epithelial repair programs [92, 93]. However, persistent type 2 signaling can activate STAT6‐linked fibroblast programs and become profibrotic [93]. In kidney disease, macrophage‐derived NGAL has been shown to promote renal fibrosis through a CCL5–Th2–IL‐4 pathway [94], and type 2 immune signaling has also been linked to diabetic kidney fibrosis [95]. Thus, Th1/Th2 remodeling should be interpreted alongside Th17/Treg imbalance as a time‐ and context‐dependent regulatory axis rather than as a uniformly pathogenic pathway.

### 4.3 Antigen‐Presenting Cell Networks and T‐Cell Amplification

Stage 2 involves both T cell polarization and remodeling of the local antigen‐presenting niche. DCs can transport kidney‐derived antigens to renal‐draining lymph nodes and present them to T cells [23, 96], whereas inflammatory macrophages provide an additional myeloid APC and cytokine‐amplifying layer in immune‐mediated kidney injury [70]. When injury persists, APC–T‐cell interactions can become organized into macrophage‐rich inflammatory niches and TLS, providing local sites for antigen presentation, cytokine production, and reciprocal activation of renal parenchymal cells [32, 97]. Human fibrosis and KPMP atlas data similarly place APC‐T‐cell amplification within immune‐stromal and immune‐parenchymal niches in diseased kidney tissue [18, 19, 20].

#### 4.3.1 Dendritic Cells and T‐Cell Priming

During the acute phase, DCs sense damage‐associated molecular patterns and help initiate T cell activation. During subacute injury, they increasingly influence T cell polarization and the Th17/Treg balance. In lupus nephritis, kidney immune‐cell mapping identifies dendritic‐cell subsets together with cytotoxic and regulatory T‐cell programs in inflamed renal tissue [98]. More generally, cDC1s specialize in antigen cross‐presentation to CD8^+^ T cells, whereas cDC2s can produce IL‐6 and IL‐23 and favor Th17 differentiation [23].

#### 4.3.2 Macrophages as Antigen‐Presenting and Cytokine‐Amplifying Partners

Macrophages provide an additional APC and cytokine‐amplifying component of the Stage 2 niche. In anti‐GBM glomerulonephritis, myeloid TLR4 loss reduces macrophage and T‐cell infiltration and shifts Th1/Th17 responses toward Treg responses, supporting macrophage control of local adaptive immunity [70]. In cisplatin‐induced renal fibrosis, CD8^+^ T cells appear close to MHC‐II^+^ macrophages, and CD4^+^ T cells form local aggregates [78]. Macrophage‐derived CCL5 can further promote CD8^+^ T‐cell recruitment in obstructive fibrosis [40], while macrophage‐lymphocyte co‐accumulation and recent single‐cell/spatial studies support the emergence of macrophage‐rich profibrotic niches after kidney injury [77, 78]. Thus, macrophage–T‐cell crosstalk links antigen presentation, cytokine amplification, and chemokine‐driven persistence, complementing DC priming during Stage 2.

#### 4.3.3 Tertiary Lymphoid Structures as Persistent APC–T‐Cell Niches

Sustained APC‐driven antigen presentation during the subacute phase can organize the immune response within renal parenchyma and promote TLS formation. In the aged mouse‐kidney injury model summarized in the cited study, TLS development followed a temporal sequence: perivascular T‐ and B‐cell aggregates appeared within weeks of continued injury, and over the next 2 to 4 months some aggregates developed distinct T‐cell zones and B‐cell follicles. Mature TLS with follicular dendritic cells and germinal‐center activity were observed after approximately 4 to 6 months in that model [32]. These mouse‐derived kinetics support placing TLS initiation within Stage 2, whereas the timing and maturation of TLS in human CKD are likely to vary by etiology, age, immune activation, and sampling interval.

TLS formation and maintenance depend on coordinated interactions between lymphocytes and stromal cells. Activated fibroblasts can provide BAFF and chemokine support within renal TLS niches [32]. The ICOS–ICOSL costimulatory axis between T and B cells is required for TLS integrity, and blockade of this pathway disrupts TLS structure [99]. Within TLS, T follicular helper (Tfh) cells and IL‐21 have central roles. IL‐21 promotes autoantibody production by germinal‐center B cells, favors Th17 differentiation while destabilizing Treg homeostasis, and directly activates renal fibroblasts through NF‐κB signaling, thereby enhancing extracellular matrix deposition [99]. In transplanted kidneys, TLS‐derived IL‐21 also drives germinal‐center responses and contributes to chronic graft dysfunction [25], suggesting a shared pathogenic mechanism in primary CKD and transplanted kidney disease.

A key feature of TLS is that they may allow inflammation to become less dependent on the initiating injury. Endogenous antigen presentation, T–B cell cooperation, and local cytokine production can sustain the process. VCAM1^+^ proximal tubular cells respond to TNF and IFN‐γ from TLS‐associated cells by upregulating TGF‐β2, CCL2, and CXCL10, thereby promoting further lymphocyte recruitment and TLS expansion [32, 100]. Current evidence suggests that TLS may be therapeutically actionable [97]. In this framework, TLS begin to emerge in Stage 2 and may later support chronic immune persistence in Stage 3. The stepwise organization of TLS maturation and function is illustrated in Figure 4.

**FIGURE 4:**
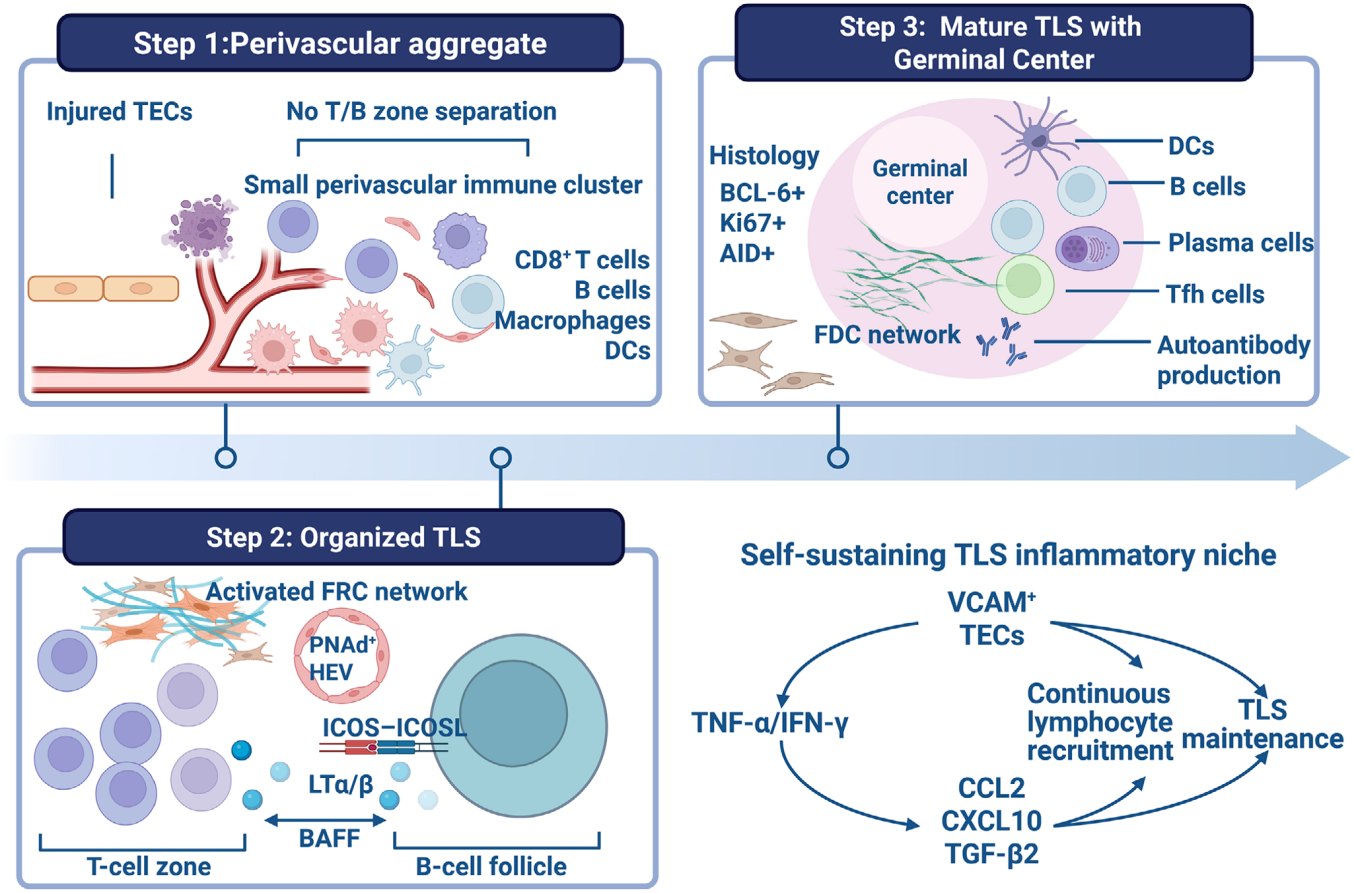
Development of tertiary lymphoid structures during renal fibrosis. Sustained kidney injury promotes perivascular immune aggregation, stromal remodeling, and gradual organization into T‐cell and B‐cell zones. With ongoing inflammation, mature TLS acquire follicular dendritic cell networks and germinal‐center‐like activity, supporting local antigen presentation, T–B‐cell cooperation, and cytokine production. These niches may help maintain inflammation after the original injury has waned. Abbreviations: FDC, follicular dendritic cell; HEV, high endothelial venule; TEC, tubular epithelial cell; TLS, tertiary lymphoid structure.

### 4.4 Metabolic Stress and Immune Dysregulation

In Stage 2, metabolic stress maintains immune dysregulation rather than simply repeating the early glycolytic switch described in Section 3.4. Glutamine metabolism becomes an important secondary energy source, and glutamine blockade with JHU083 reduces T‐cell activation and protects against ischemic and nephrotoxic injury [101]. At the same time, weakening of Treg fatty acid oxidation and AMP‐activated protein kinase (AMPK)/SIRT1 support may impair suppressive function and favor effector expansion [30].

Lipid and amino acid metabolism also influence fibrotic immunity. In glomerular mesangial cells, palmitic acid drives progression from lipotoxic injury to fibrosis through CD36/TRPC6/NFAT2 signaling [102]. In uremia, indoxyl sulfate (IS) promotes expansion of CD4^+^/CD28^+^ T cells through aryl hydrocarbon receptor (AhR) signaling in patients with end‐stage kidney disease (ESKD) and amplifies chronic inflammation by inducing trained immunity in monocytes through ALOX5‐dependent arachidonic acid metabolism [103]. In parallel, IDO1‐mediated tryptophan depletion and kynurenine accumulation promote AhR‐dependent upregulation of PD‐1 on CD8^+^ T cells, linking amino acid metabolism to immune checkpoint regulation [104]. During IRI, PFKFB3‐driven lactate accumulation can induce H4K12 lactylation and activate NF‐κB‐dependent profibrotic gene programs, while renin has been reported to enhance NLRP3 inflammasome activation [105]. More broadly, hypoxia, nutrient deprivation, and metabolic waste within fibrotic tissue impose chronic stress on infiltrating T cells. These conditions may promote HIF‐1α‐dependent stress responses, mitochondrial dysfunction, and progressive T‐cell impairment, as discussed in Sections 5.1 and 5.5 [106, 107, 108].

Together, these findings suggest that glycolysis, lipid metabolism, and mitochondrial dysfunction form connected metabolic transitions. Early glycolytic flux and lactate production support effector and chromatin‐permissive programs [101, 105], lipid and amino‐acid stress later reshapes regulatory balance and checkpoint tone [56, 106, 107], and late mitochondrial dysfunction promotes low‐renewal, ROS‐rich, exhaustion‐prone states [107, 108]. This feed‐forward pattern may explain how early metabolic reprogramming evolves into persistent immune activation rather than resolution.

### 4.5 Signaling Hubs in Stage 2

Several converging pathways stabilize the Th17/Treg imbalance in Stage 2. One of the most important is SGK1, which links extracellular sodium to T‐cell fate. Increased extracellular Na^+^ induces SGK1‐related Th17 programming in high‐salt or salt‐sensitive renal injury models [67, 68]. In DKD, SGK1 inhibition by dapagliflozin has been linked to restoration of Th17/Treg balance and attenuation of renal injury [84]. In Tregs, SGK1‐mediated FOXO1 phosphorylation drives nuclear exclusion of FOXO1, inhibits Foxp3 transcription, and selectively weakens the suppressive function of thymus‐derived natural Tregs under high‐salt conditions, with more limited effects on TGF‐β‐induced peripheral Tregs [109, 110]. SGK1 can also regulate TGF‐β/Smad signaling through NEDD4L/Smad turnover [111], and kidney fibrosis studies link SGK1 to EMT and extracellular matrix deposition through SGK1‐ or FOSL2/SGK1‐related pathways [112, 113]. Thus, SGK1/high‐salt signaling is best viewed as a context‐dependent amplifier, especially in high‐salt post‐AKI, salt‐sensitive hypertensive nephropathy, and selected DKD settings. In these contexts, dietary sodium restriction may help reduce immune dysregulation and slow fibrotic progression.

A second pathway is Orai1/calcineurin/NFAT signaling. By sustaining SOCE, Orai1 maintains calcineurin activity and NFAT nuclear localization. In the nucleus, NFATc1 cooperates with STAT3 activated by IL‐6 and IL‐21, promoting Rorc and Il17a expression and strengthening Th17 polarization [66, 114]. In models of progression from AKI to CKD, Orai1 blockade reduces Th17 infiltration and attenuates interstitial fibrosis. Urinary SOCE activity has also been proposed as a marker of T cell activation [66].

The NLRP3 inflammasome provides another relay that reinforces immune imbalance. Injured TECs and damaged renal tissue release or expose DAMPs, including extracellular ATP, uric acid‐related crystal signals, and mitochondrial DNA, which can activate NLRP3 and promote caspase‐1‐dependent maturation of IL‐1β and IL‐18 in renal injury [115, 116]. In obstructed kidneys, renal dendritic cells and monocytes produce IL‐1, which enhances intrarenal Th17 activation and is reduced by IL‐1R deficiency or IL‐1R blockade [117]. Mechanistically, IL‐1β cooperates with IL‐6/IL‐23 to promote STAT3/RORγt‐associated Th17 programming [118], while IL‐1R/IRAK1 signaling can limit TGF‐β‐driven Foxp3 induction and shift CD4^+^ T‐cell differentiation toward IL‐17‐producing programs [119, 120]. Thus, NLRP3–IL‐1β signaling links injured renal tissue to Th17/Treg imbalance in Stage 2.

mTOR signaling also shapes lineage decisions in this setting. Consistent with established T‐cell metabolism principles, mTORC1 supports glycolytic effector differentiation and can oppose Foxp3 induction, whereas mTORC2 influences T‐cell differentiation and Treg function in a context‐dependent manner [53]. This complexity suggests that broad mTOR inhibition may have mixed effects on Th17/Treg balance, whereas more selective targeting of mTORC1‐linked effector metabolism may be better suited to restoring immune balance. Together, these pathways sustain maladaptive T‐cell states and promote chronic inflammation.

## 5 Stage 3: Exhaustion and Tissue Residency in Advanced Fibrosis

As immune and metabolic stress accumulate, fibrosis may become less reversible and increasingly dependent on local immune niches. In Stage 3, prolonged antigen exposure reinforces TOX‐associated exhaustion programs, while TGF‐β, IL‐10, uremic metabolites, and mitochondrial dysfunction reshape the remaining T cell response. The result is an accumulation of remodeled T cell populations, including TEX and TRM, that can sustain chronic profibrotic signaling.

### 5.1 T‐Cell Exhaustion in Late Fibrosis

In late renal fibrosis, T‐cell exhaustion appears across kidney failure, transplant, and chronic injury datasets. It develops under sustained antigen exposure, including alloantigens, autoantigens, and latent viral antigens, together with suppressive cues such as TGF‐β, IL‐10, prostaglandins, and uremic metabolites [10, 11].

Single‐cell and mass cytometry studies have defined TEX as heterogeneous rather than a single terminal endpoint. In chronic infection and cancer, TCF‐1^+^ progenitor‐like TEX retain stem‐like renewal capacity and can supply the proliferative response after PD‐1 blockade [121, 122]. Broader exhaustion studies organize CD8^+^ TEX along progenitor, intermediate, and terminal states, with progressive loss of self‐renewal and effector function [123, 124]. In kidney disease, current evidence is more limited but supports the clinical relevance of exhausted CD8^+^ T‐cell phenotypes, particularly in transplantation, where coexpression of PD‐1, TIGIT, 2B4, 4‐1BB, and TIM‐3 has been associated with renal outcomes [10, 125]. Mechanistically, chronic antigen/TCR stimulation promotes TOX/NR4A‐dependent transcriptional remodeling and progression toward more fixed exhaustion states [126, 127]. Together, TCF‐1‐associated progenitor potential and TOX/NR4A‐mediated transcriptional remodeling provide a cautious framework for interpreting TEX heterogeneity in late renal fibrosis until longitudinal kidney‐specific validation becomes available.

Functionally, TEX have a paradoxical role. Reduced IL‐2, IFN‐γ, and TNF‐α production, together with lower proliferation and cytotoxicity, may limit acute immunopathology. In kidney transplant recipients, higher proportions of exhausted CD4^+^ and CD8^+^ T cells 6 months after transplantation were associated with less progressive interstitial fibrosis, with the strongest inverse association observed in the PD‐1^+^/TIGIT^+^/2B4^+^/4‐1BB^+^/TIM‐3^+^ CD8^+^ subset [10]. At the same time, exhaustion may permit chronic disease by weakening clearance of senescent cells or latent viral infection, including cytomegalovirus and BK virus, thereby maintaining antigenic stimulation and inflammation.

This dual role should be interpreted as an adaptive‐to‐maladaptive continuum rather than a simple protective or pathogenic label. Partial exhaustion may act as a regulatory brake that limits cytokine‐driven tissue injury, whereas fixed terminal exhaustion can impair proliferative renewal, antiviral effector function, and antitumor surveillance [123, 124]. In kidney transplant recipients, BK polyomavirus clearance correlates with the exhaustion state and T‐cell receptor repertoire of BKV‐specific T cells, supporting the concern that late TEX may compromise immune surveillance in susceptible hosts [128]. Thus, late‐stage TEX may restrain acute immunopathology while allowing latent viruses, senescent cells, or emerging malignant clones to persist [129].

Some exhausted subsets retain pathogenic activity. Terminally exhausted TIM‐3^+^ CD8^+^ T cells can still secrete granzyme B and persist in fibrotic lesions such as inflammatory interstitial fibrosis and tubular atrophy (i‐IFTA) in transplanted kidneys. In kidney transplant recipients, peripheral cytotoxic T lymphocytes are reduced, whereas granzyme B expression within the graft is increased and correlates with proteinuria and local transcript abundance [130]. Mechanistically, extracellular granzyme B can cleave matrix‐associated proteins, including decorin and biglycan, as well as soluble betaglycan, releasing active TGF‐β1 and supporting downstream profibrotic signaling [69]. It can also promote EMT through both TGF‐β‐dependent and TGF‐β‐independent pathways [131, 132].

Checkpoint signaling is remodeled in the late fibrotic microenvironment. IL‐6‐induced MMP‐2 can cleave PD‐1 from the T cell surface to generate soluble PD‐1 (sPD‐1), which binds PD‐L1 on renal tubular epithelial cells and partially restores T cell effector function [133]. In IgA nephropathy, serum sPD‐1 predicts interstitial fibrosis severity and renal functional decline. In the UUO model, PD‐L1 fusion protein restores coinhibitory signaling and reverses this effect [133]. A 2025 multicohort study further showed that serum sPD‐L1 rises with CKD progression and increases after dialysis [134]. These findings suggest that shedding of checkpoint receptors and ligands may progressively weaken checkpoint‐mediated restraint. The progression from progenitor to terminally exhausted CD8^+^ T cells is summarized in Figure 5.

**FIGURE 5:**
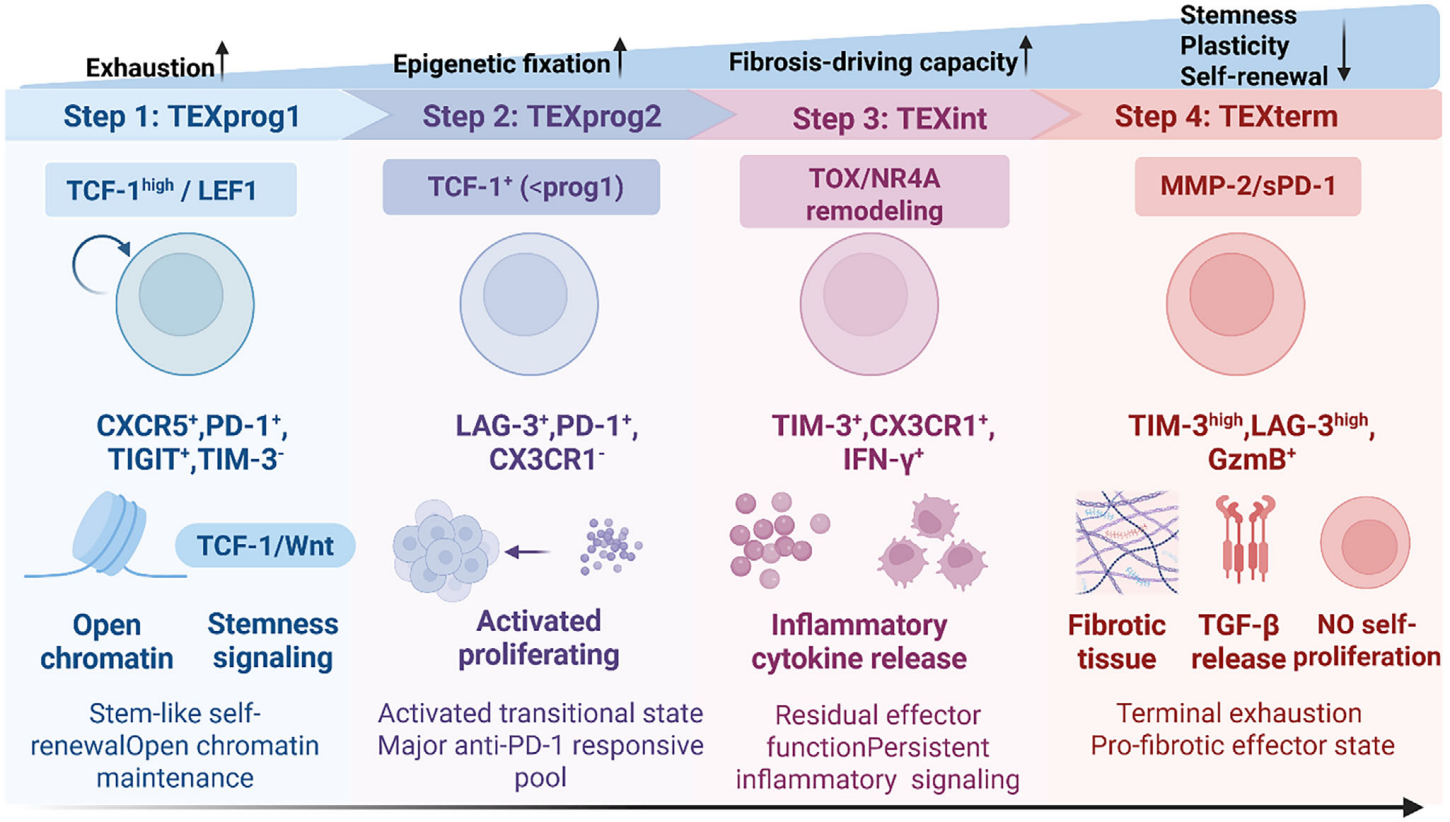
Continuum of CD8^+^ T‐cell exhaustion in chronic renal fibrosis. Sustained antigen exposure and metabolic stress move CD8^+^ T cells from progenitor exhausted states toward intermediate and terminally exhausted phenotypes. Progenitor exhausted cells retain partial self‐renewal and may remain responsive to checkpoint modulation, whereas terminally exhausted cells have limited proliferative capacity but may preserve selected pathogenic functions in fibrotic tissue. This late‐stage state may restrain acute immunopathology but can also weaken antiviral and antitumor immune surveillance. TOX/NR4A‐associated transcriptional remodeling helps stabilize this trajectory. Abbreviations: NR4A, nuclear receptor subfamily 4 group A; sPD‐1, soluble programmed cell death protein 1; TCF‐1, T cell factor 1; TEX, exhausted T cell; TOX, thymocyte selection‐associated high mobility group box protein.

### 5.2 Tissue‐Resident Memory T Cells in Fibrotic Niches

In advanced fibrosis and immune‐mediated nephritis, effector memory T cells can differentiate into TRM‐like states and persist in kidney niches [27, 28]. Renal TRM differ from those in epithelial barrier tissues. They often lack CD103 and instead rely on VLA‐1 and LFA‐1 for tissue retention, while CD69, CD38, and CD39 appear to be more informative phenotypic markers [125, 135].

Renal studies implicate the CXCL16–CXCR6 axis in kidney inflammation, fibrosis, and TRM‐rich glomerular contexts [43], while nonrenal dermal CD8^+^ TRM work provides direct evidence that CXCR6 can support long‐term tissue residence [136]. In renal fibrosis, this axis is therefore best viewed as a kidney‐relevant but incompletely validated retention pathway. In kidney disease models, angiotensin II and endothelin‐1 released by injured parenchymal cells can increase IL‐15 and promote CD8^+^ TRM expansion through CD122, driving podocyte injury and glomerulosclerosis [28]. Antagonism of AT1 and ETA receptor signaling, for example with sparsentan, may therefore inhibit the angiotensin II/endothelin‐1‐IL‐15‐TRM axis and reduce TRM‐mediated glomerular injury [28].

TRM can be activated through antigen‐dependent and antigen‐independent pathways. MHC class I‐mediated antigen presentation sustains IFN‐γ and perforin secretion. In parallel, IL‐12, IL‐15, and IL‐18 can drive TCR‐independent bystander activation in chronically inflamed tissue, allowing TRM cells to remain active even without cognate antigen [27, 28]. Within renal CD8^+^ TRM, NLRP3 inflammasome activation enhances differentiation and pathogenicity through the caspase‐1‐GSDMD‐IL‐1β pathway. The NLRP3 inhibitor MCC950 reduces TRM‐mediated kidney injury in a lupus model, supporting inflammasome activity as a driver of TRM persistence [137]. Blockade of IL‐15/CD122 signaling also suppresses TRM cytotoxicity and delays fibrosis [27, 28]. TRM function also depends on antigen specificity: CD8^+^ T cells directed against renal autoantigens can differentiate into TRM with both activation and dysfunction features and persist in the kidney, whereas circulating memory T cells of the same specificity disappear much more rapidly [27].

Not all TRM programs should be interpreted as pathogenic. In broader tissue studies, resident or resident‐like T cells can support local surveillance, resolution of inflammation, epithelial growth, and tissue remodeling [138, 139]. In the kidney, direct evidence for reparative TRM remains limited; however, the repair‐promoting effects of Tregs and double‐negative T cells after ischemic AKI suggest that protective kidney‐adapted T‐cell programs should be distinguished from cytotoxic or inflammasome‐high TRM in future studies [86, 140]. Human kidney profiling studies support spatial immune niches and resident‐like T‐cell programs in kidney tissue [29], while renal autoimmune studies document TRM accumulation in lupus nephritis and related immune‐mediated nephritides [125]. Experimental kidney models further link CD8^+^ TRM to podocyte injury and glomerulosclerosis [28]. However, whether the same TRM program dominates common metabolic or hypertensive forms of CKD remains less well defined. The survival, retention, and reactivation signals that sustain renal TRM are summarized in Figure 6.

**FIGURE 6:**
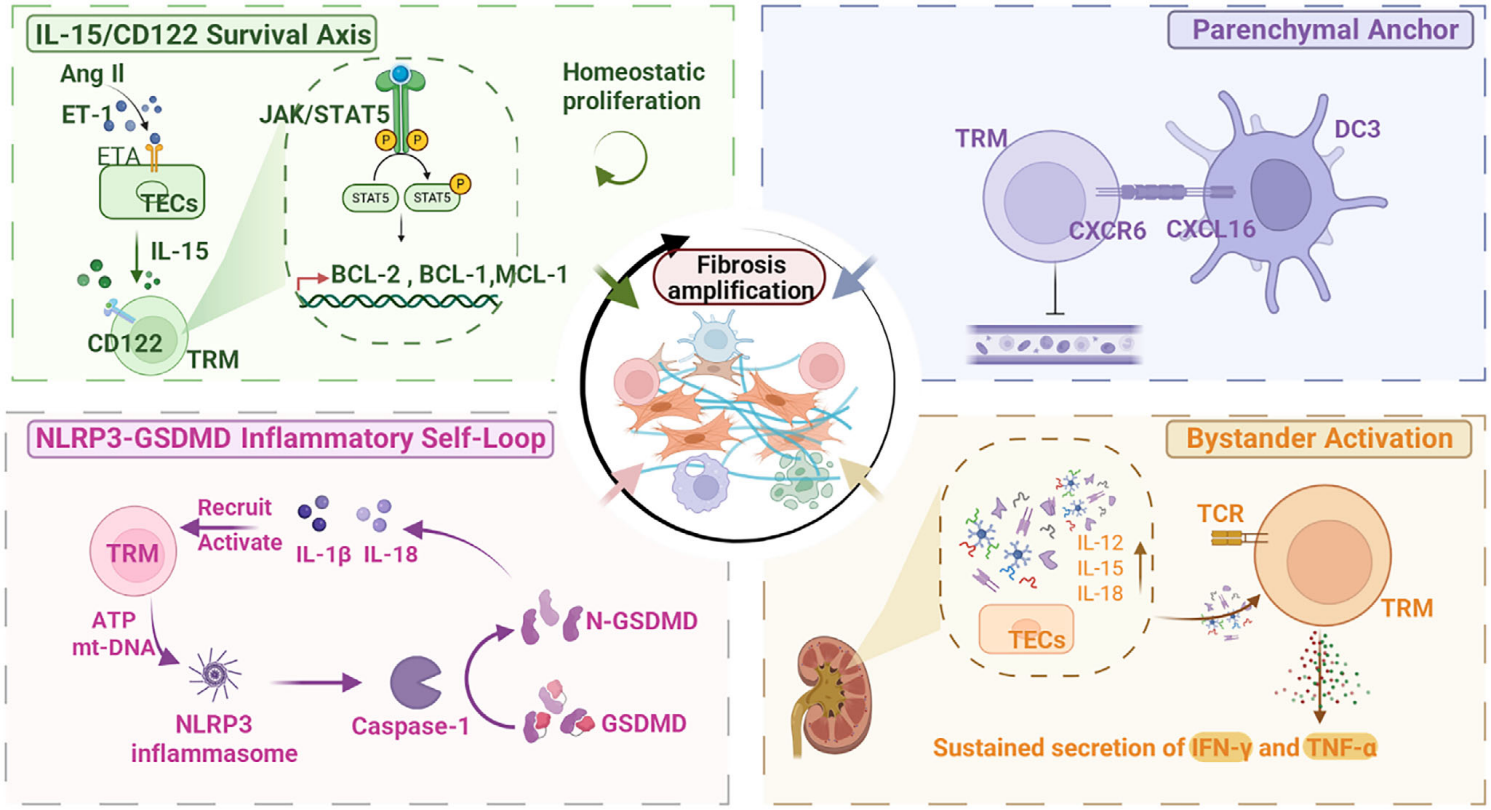
Mechanisms that sustain TRM in advanced renal fibrosis. TRM persist in chronically injured kidney tissue through survival signals, stromal retention cues, bystander cytokine activation, and innate immune amplification. IL‐15/CD122 signaling, CXCL16–CXCR6 interactions, local antigen presentation, and inflammasome activation may reinforce TRM maintenance and pathogenic reactivation. Depending on context, these pathways may support local surveillance or sustain profibrotic inflammation. Abbreviation: TRM, tissue‐resident memory T cell.

### 5.3 Senescence, SASP, and T‐Cell Feedback

T‐cell senescence and exhaustion overlap in late fibrosis, but they are not equivalent. Exhaustion is mainly driven by persistent antigen/TCR signaling and TOX/NR4A‐associated checkpoint programs, whereas senescence is linked to aging, repeated proliferation, DNA/telomere damage, mitochondrial stress, uremic inflammation, CD28/CD27 loss, CD57/KLRG1 acquisition, and senescence‐associated secretory phenotype (SASP)‐like cytokine output [123, 124, 132]. Clinically, TEX mainly reflects checkpoint restraint and immune‐surveillance trade‐offs, whereas senescent T cells indicate impaired immune renewal and chronic inflammatory remodeling; in CKD, reduced thymic output and expansion of CD4^+^CD28^−^ T cells have been associated with adverse renal outcomes [141].

In aging and advanced kidney disease, senescent cells and the SASP create a profibrotic tissue environment. Renal single‐cell analysis supports a profibrotic and proinflammatory senescent epithelial program after kidney injury [142]. Senescent cells evade immune clearance through several mechanisms. In broader senescence models, senescent fibroblasts and epithelial cells upregulate CD47 and suppress macrophage‐mediated efferocytosis through the CD47–SIRPα/SHP‐1 axis [143]. They also increase PD‐L1 expression in a SASP‐dependent manner, reducing susceptibility to clearance by PD‐1‐expressing cytotoxic CD8^+^ T cells [144]. Single‐cell studies show that PD‐L1 expression in senescent cells correlates with SASP intensity. In aged mice, anti‐PD‐1 treatment reduced senescent cell burden and improved senescence‐associated phenotypes, linking this process to the exhaustion mechanisms discussed in Section 5.1 [145]. SASP factors can also upregulate PD‐L1 through JAK‐STAT signaling and extend immunosuppressive effects to neighboring nonsenescent cells [146].

Together, these findings support a senescence–immunity model for renal fibrosis, in which CKD‐associated immune aging and senescent T‐cell–TEC feedback reinforce chronic inflammation. Whether checkpoint‐mediated clearance of senescent cells can be harnessed therapeutically in renal fibrosis remains to be tested.

The senescent microenvironment also feeds back on T cells. After unresolved IRI, tubular GM‐CSF induces macrophage MCP‐1/CCR2 signaling and supports persistent macrophage, dendritic‐cell, and T‐cell accumulation during fibrotic repair [147]. Senescent T cells can further aggravate renal epithelial injury: in an angiotensin II model, aged/senescent T cells produced more IFN‐γ, and conditioned medium from these cells induced oxidative stress and inflammatory responses in TECs; IFN‐γ neutralization blunted these effects [148]. These interactions create a loop in which SASP signals and injured‐tubule chemokines recruit or activate immune cells, while T‐cell‐derived mediators reinforce TEC senescence, immune evasion, and fibrosis. This reciprocal feedback loop between SASP signaling and T cells is summarized in Figure 7.

**FIGURE 7:**
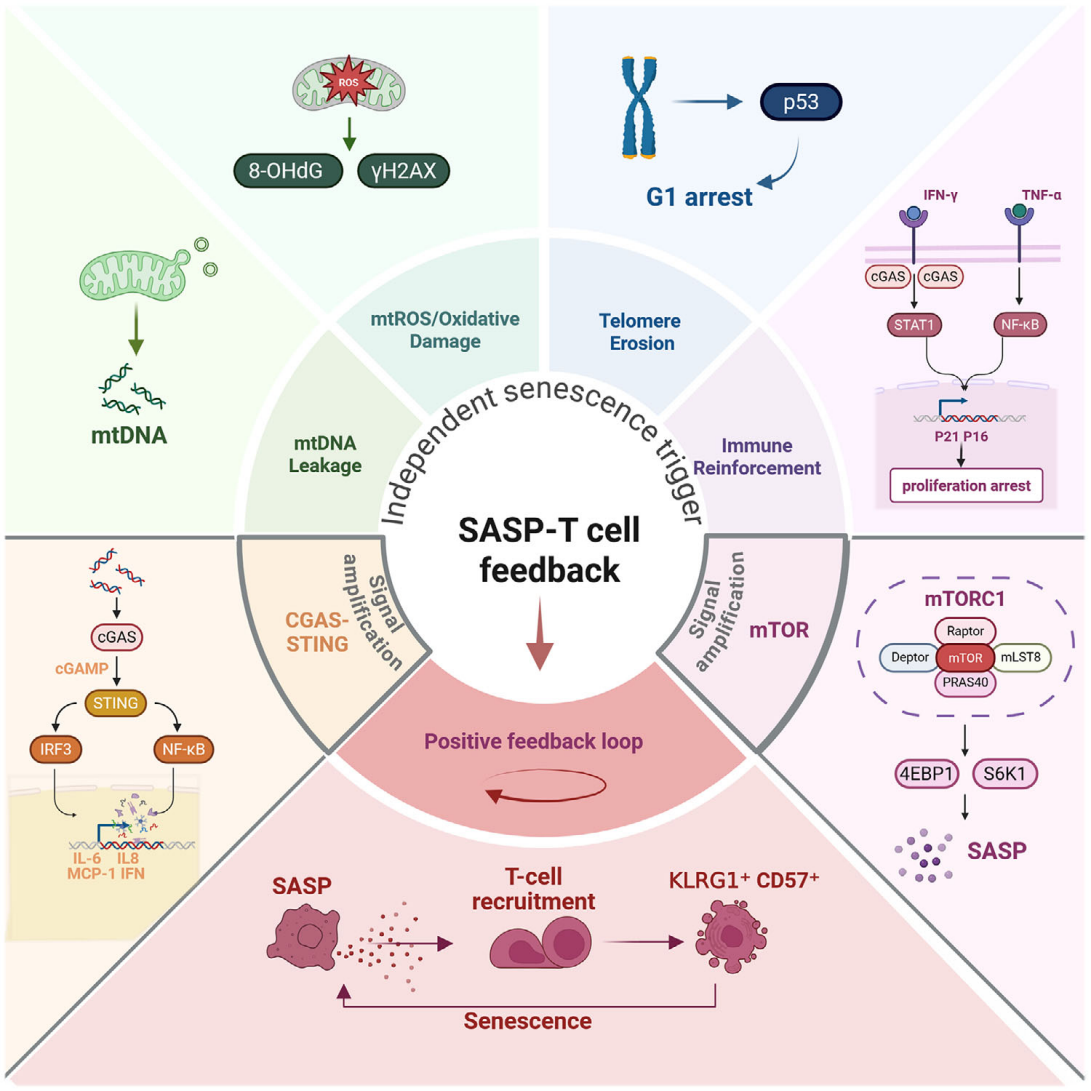
Feedback between SASP signaling and T cells in late renal fibrosis. Oxidative stress, mitochondrial dysfunction, telomere instability, and chronic inflammation promote tubular epithelial cell senescence. Senescent cells generate a SASP‐rich environment that recruits and activates T cells, while T‐cell‐derived inflammatory mediators reinforce tubular senescence and profibrotic signaling. This reciprocal loop links immune remodeling to irreversible tissue injury in advanced kidney disease. Abbreviations: cGAS, cyclic GMP–AMP synthase; mtROS, mitochondrial reactive oxygen species; SASP, senescence‐associated secretory phenotype; STING, stimulator of interferon genes; TEC, tubular epithelial cell.

mTOR signaling is an important regulator of the inflammation–senescence cycle. Rapamycin downregulates PD‐L1 in senescent cells in broader senescence models [146], and low‐dose rapamycin after experimental kidney transplantation reduces p16^+^ senescent cells, SASP cytokines, monocyte/macrophage and CD8^+^ T‐cell infiltration, and early fibrotic injury [149]. Senolytic CAR T cells targeting uPAR can eliminate senescent cells in vivo in models of liver fibrosis and age‐related metabolic dysfunction [150, 151]. These studies provide cross‐organ proof of concept, but renal translation will require evidence that uPAR marks relevant senescent or profibrotic renal‐cell populations and can be targeted without kidney‐specific toxicity.

### 5.4 Metabolic Failure in Late‐Stage T Cells

In late fibrosis, T cell metabolism progressively fails. In terminally exhausted T cells, downregulation of peroxisome proliferator‐activated receptor gamma coactivator 1‐alpha (PGC‐1α) and mitochondrial transcription factor A (TFAM) impairs mitochondrial biogenesis, reduces mitochondrial mass and membrane potential, and is associated with mitochondrial fragmentation and inadequate OXPHOS. The result is persistent ATP insufficiency [145]. Mitochondrial dysfunction also promotes ROS accumulation, which stabilizes HIF‐1α, shifts metabolism toward aerobic glycolysis, increases inhibitory receptor expression, and accelerates progression from precursor states to terminal exhaustion [145].

Late metabolic dysfunction may extend beyond CD8^+^ exhausted T cells. In Tregs, LKB1 supports mitochondrial integrity, fatty acid oxidation, and suppressive function; disruption of this axis reduces ATP production and may weaken regulatory control [106]. These findings suggest that energetic insufficiency across several T cell populations can worsen immune dysregulation in advanced fibrosis.

The surrounding tissue contributes to this process. Within the fibrotic tubular microenvironment, TECs amplify metabolic inflammation. In cisplatin nephrotoxicity, TIM‐3 appears to have a protective role: loss of TIM‐3 increases mitochondrial ROS generation and 8‐OHdG accumulation, activates NF‐κB p65, and promotes tubular apoptosis and inflammation. These effects can be reversed by NF‐κB inhibition [152].

Oxidative stress and aberrant lipid metabolism within the immune–renal microenvironment may also contribute to fibroblast activation and tubular toxicity. Circulating uremic metabolites, including indoxyl sulfate, further aggravate T‐cell metabolic dysfunction, creating a reinforcing cycle between immune metabolic failure and tissue fibrosis [30]. This disequilibrium may impair T‐cell surveillance and clearance of senescent cells while strengthening SASP‐driven profibrotic amplification, as discussed in Section 5.3. In peripheral blood mononuclear cells from patients with ESKD, basal respiration, ATP production, maximal respiration, and spare respiratory capacity are reduced, accompanied by lower AMPKα activity, lower PGC‐1α expression, and abnormal mitochondrial fission‐fusion dynamics [153]. These observations provide a rationale for NAD^+^ precursor supplementation and mitochondria‐targeted antioxidant strategies in appropriately staged disease.

### 5.5 Integrated Signaling Networks in Advanced Fibrosis

In advanced renal fibrosis, several pathways converge to translate immune dysfunction into structural scarring. Among them, IFN‐γ/STAT1 and TGF‐β/Smad3 act as major effector‐output hubs. IFN‐γ is a key T‐cell effector cytokine in hypertensive renal injury and can also be increased by senescent T cells in angiotensin II‐associated target‐organ damage [148, 154]. Through JAK1/2–STAT1 signaling, IFN‐γ can induce antigen‐presentation and inflammatory programs in renal epithelial cells, including MHC molecules and chemokines; EMT‐associated factors such as Twist1 and Snail should be interpreted as downstream remodeling markers within this inflammatory–fibrotic environment [38, 154]. STAT1 also promotes M1 macrophage polarization, while IL‐12 helps sustain IFN‐γ production.

At the same time, IFN‐γ/STAT1 signaling induces Smad7 through a STAT1‐binding element in the Smad7 promoter [155]. Smad7 competes with Smad3 for binding to the type I TGF‐β receptor and promotes ubiquitin‐mediated degradation of the receptor complex, thereby limiting fibrosis [155]. This restraint is progressively lost in late disease. Smad3 induces Smurf1 and Smurf2, which polyubiquitinate Smad7 and accelerate proteasomal degradation [63]. Smad3‐driven miR‐21 also targets the 3′ UTR of Smad7 and reduces Smad7 abundance post‐transcriptionally [64]. As T‐cell exhaustion progresses and IFN‐γ output may decline, the antifibrotic brake imposed by Smad7 may weaken, leaving TGF‐β/Smad3 signaling increasingly dominant [123, 155].

The TGF‐β/Smad pathway is a major route to extracellular matrix deposition. Among receptor‐activated Smads, Smad3 is a key transcriptional regulator of profibrotic genes, whereas Smad2 and Smad7 can be protective in some settings [63]. In the late renal microenvironment, Smad3 activation is not fully dependent on TGF‐β1; it can also be triggered through MAPK signaling downstream of angiotensin II, advanced glycation end products, and C‐reactive protein [63]. This may help explain why upstream TGF‐β1 blockade alone has limited antifibrotic efficacy. TGF‐β bioavailability may also increase when extracellular granzyme B cleaves decorin, biglycan, and soluble betaglycan, releasing active TGF‐β1 and inducing SMAD3 signaling [69].

Persistent fibrosis may also involve direct crosstalk between T cells and fibroblasts. In inflammatory fibrotic tissues, activated fibroblasts can express antigen‐presentation molecules such as MHC II, HLA‐DRA, and CD74, and may help sustain local CD4^+^ T‐cell activation through cell‐contact signals [156, 157]. Kidney‐specific studies increasingly support this immune–stromal communication model: human kidney fibrosis atlases identify disease‐associated myofibroblast programs and spatial immune–stromal niches [20, 29], and single‐cell analysis of renal tubulointerstitial fibrosis shows increasing fibroblast–immunoinflammatory cell communication during progression [158]. These data support a feed‐forward loop spanning Stages 2 and 3 in which inflammatory fibroblasts retain and reactivate T cells, while T‐cell‐derived IFN‐γ, TNF‐α, IL‐17, and granzyme B further promote fibroblast activation, extracellular matrix remodeling, and TGF‐β release.

Mitochondrial stress further reinforces this output layer. In renal fibrosis, mitochondrial dysfunction is linked to ROS‐driven inflammatory and profibrotic programs [108], and in cisplatin nephrotoxicity TIM‐3 loss increases mitochondrial ROS and NF‐κB p65 activation [152]. Progressive loss of AMPK activity also relieves inhibition of mTORC1, allowing T cell effector programs and fibroblast biosynthetic activity to continue. In parallel, cytoplasmic DNA released from damaged organelles can activate the cyclic GMP–AMP synthase–stimulator of interferon genes (cGAS–STING) pathway and promote interferon regulatory factor 3 (IRF3)‐dependent interferons and NF‐κB‐dependent cytokines. The cGAS–STING pathway has been implicated in renal inflammation and fibrosis, but this kidney‐fibrosis evidence should be distinguished from broader organ‐specific STING biology [159]. Mitochondrial injury, inflammatory activation, and fibrotic signaling therefore reinforce one another in late disease.

## 6 Therapeutic Strategies Targeting T Cells Across Disease Stages

Candidate therapeutic opportunities across stages are summarized in Table 3 by disease stage, dominant immune program, and evidence scope. Delivery platforms and cell‐based therapies are placed within the stage where they are most mechanistically relevant. The evidence‐scope categories used in Table 3 are defined in the accompanying table note.

**TABLE 3:** Candidate therapeutic opportunities targeting T cells across stages of renal fibrosis.

Stage	Therapeutic category	Representative approach	Target pathway/mechanism	Expected benefit	Evidence level	Refs
Stage 1	Chemokine‐axis modulation	CXCL9/CXCL10–CXCR3 targeting	CXCL9/CXCL10–CXCR3 axis	Limit CXCR3^+^ Th1 and effector CD8^+^ T‐cell recruitment; potential antifibrotic effects of CXCL10 remain context‐dependent	Direct renal fibrosis/AKI models; no CKD trial	[37, 38, 160]
Stage 1	Chemokine‐axis modulation	CCL5–CCR5 blockade	NET–macrophage–CCL5 relay	Interrupt NET–macrophage CCL5 signaling and reduce CD8^+^ T‐cell recruitment	Direct renal fibrosis model; preclinical	[40, 75]
Stage 1	Targeted delivery	VCAM‐1/ICAM‐1‐targeted engineered vesicles (Foe‐TEVs)	Adhesion molecule‐guided targeting	Deliver immunomodulatory cargo to inflamed renal tissue and reduce alloreactive T‐cell activation	Direct renal transplant model; preclinical delivery	[161]
Stage 1	Metabolic modulation	mTOR/AMPK modulation; QSG‐related PI3K/Akt/mTOR targeting	PI3K/Akt/mTOR/AMPK pathways	Restrain activation‐associated glycolysis and translation; renal T‐cell specificity remains uncertain	Direct renal fibrosis model; T‐cell mechanism indirect	[30, 53]
Stage 1	Glutamine metabolism	JHU083 (DON prodrug)	Glutamine‐dependent metabolism	Suppress glutamine‐dependent T‐cell activation and inflammatory expansion during AKI	Direct renal AKI model; fibrosis reversal untested	[101]
Stage 1	Glycolysis–lactylation pathway	PFKFB3 inhibition (3PO)	PFKFB3–lactate–H4K12 lactylation axis	Reduce lactate production, H4K12 lactylation, and NF‐κB activation	Direct renal fibrosis model; T‐cell indirect	[105]
Stage 2	Th17/Treg modulation	Dapagliflozin (SGLT2 inhibitor)	SGK1/Foxo1–IL‐23R signaling	Improve CKD outcomes; SGK1‐linked Th17/Treg modulation remains mainly preclinical	Clinical CKD outcome; T‐cell mechanism preclinical	[84, 162]
Stage 2	SOCE/Orai1 inhibition	YM58483; Orai1 blockade	SOCE–NFAT–RORγt pathway	Reduce IL‐17A production and Th17‐associated fibrosis	Direct AKI‐to‐CKD model + human AKI blood	[66]
Stage 2	Costimulation blockade	Abatacept (CTLA4‐Ig)	B7–CD28 signaling	Reduce renal T‐cell infiltration, Th17 cytokines, and proteinuric injury	Direct renal preclinical; clinical use nonrenal	[163, 164]
Stage 2	Treg expansion/support	CD28 superagonists; low‐dose IL‐2; VDR‐supportive approaches	Treg maintenance pathways	Expand functional Tregs and restore immune restraint after AKI/IRI or transplantation	Direct renal IRI/transplant; broad fibrosis indirect	[86, 165, 166]
Stage 2	Epigenetic and plasticity modulation	HDAC inhibition; miPEP31	FOXP3–IL‐17^+^ plasticity regulation	Limit pathogenic T‐cell plasticity and promote Treg differentiation	Renal preclinical/indirect; no clinical validation	[31, 167]
Stage 2	TLS/Tfh‐axis disruption	Anti‐CD4; anti‐CD153 targeting; ICOS–ICOSL/IL‐21 blockade	TLS maintenance and Tfh signaling	Disrupt TLS maintenance, T–B cooperation, Tfh‐derived IL‐21 signaling, and fibroblast activation	Renal preclinical + TLS observation; no trial	[25, 32, 97, 99]
Stage 2	DC targeting	FLT3 inhibitor AC220	CD103^+^ DC‐mediated T‐cell priming	Deplete pathogenic CD103^+^ DCs and suppress CD8^+^ T‐cell activation upstream of TLS formation	Direct renal preclinical + human FSGS tissue	[79]
Stage 3	Checkpoint remodeling	PD‐1/PD‐L1 restoration; sPD‐1 targeting	PD‐1/PD‐L1 checkpoint axis	Counter soluble checkpoint disruption that reactivates T cells and promotes profibrotic signaling	Direct renal model + human biomarker	[133, 134]
Stage 3	Senescence/SASP control	Rapamycin; navitoclax	Senescence‐associated inflammatory pathways	Reduce senescent cell burden and SASP‐mediated immune recruitment; fibrosis‐specific clinical evidence remains limited	Direct renal senescence models; no reversal trial	[142, 149, 168]
Stage 3	Metabolic restoration	Nicotinamide/NAD^+^ precursors	Mitochondrial metabolic pathways	Improve mitochondrial fitness and tubular inflammation; direct rescue of exhausted renal T‐cell metabolism remains indirect	Direct renal preclinical; T‐cell rescue indirect	[169]
Stage 3	TRM targeting	Anti‐IL‐15/CD122; MCC950	IL‐15/CD122 and NLRP3–caspase‐1–GSDMD–IL‐1β axis	Suppress TRM maintenance and pathogenicity	Direct renal/glomerular models; no clinical TRM therapy	[28, 137]
Stage 3	Senolytic cell therapy	Anti‐uPAR CAR T cells (cross‐organ); PDGFRβ/FAP‐targeted CAR T cells (renal preclinical)	Senescent‐cell or ECM‐producing‐cell targeting	Explore clearance of senescent cells or remodeling of scar‐forming cells; kidney‐directed CAR T‐cell therapy remains preclinical	uPAR cross‐organ proof‐of‐concept; PDGFR/FAP direct renal preclinical; no clinical data	[29, 98, 170, 171]
All stages	Engineered vesicle/cell‐free immunomodulation	Treg‐derived Foe‐TEVs; MSC/PMSC‐derived extracellular vesicles	CTLA4/IL‐10 delivery and immune reprogramming	Deliver immunomodulatory cargo and reshape local immune responses	Direct renal/transplant preclinical; delivery platform	[161, 172, 173]
All stages	Immune‐reset cell therapy	CD19 CAR T cells	B‐cell depletion	Eliminate autoreactive B cells and reset adaptive immunity; currently validated mainly in SLE/LN and ANCA‐associated disease	Clinical autoimmune/LN evidence + renal preclinical; nonimmune CKD untested	[174, 175, 176, 177, 178]
All stages	Natural products and TCM	QSG; Jin‐Gui‐Shen‐Qi‐Wan; cordyceps proteins	PI3K/Akt/mTOR, MHC II/CD4^+^ T‐cell polarization, Th1/T‐cell infiltration pathways	Modulate immune‐metabolic pathways in selected contexts; clinical maturity is heterogeneous	Mixed: selected CKD clinical + renal preclinical; formula‐specific	[65, 90, 179, 180]

Note: Evidence scope indicates the closest tested context for each strategy, including direct renal fibrosis or AKI‐to‐CKD models, clinical evidence in kidney disease, broad CKD outcome evidence with indirect immune mechanisms, or cross‐organ proof‐of‐concept data. Abbreviations: AKI, acute kidney injury; CAR, chimeric antigen receptor; CKD, chronic kidney disease; FAP, fibroblast activation protein; Foe‐TEV, Foxp3‐engineered Treg‐derived extracellular vesicle; FSGS, focal segmental glomerulosclerosis; IRI, ischemia–reperfusion injury; LN, lupus nephritis; MSC, mesenchymal stromal cell; PDGFRβ, platelet‐derived growth factor receptor beta; PMSC, placental mesenchymal stromal cell; QSG, Qingshen Granules; SASP, senescence‐associated secretory phenotype; SOCE, store‐operated calcium entry; TCM, traditional Chinese medicine; TLS, tertiary lymphoid structure; uPAR, urokinase plasminogen activator receptor.

Therapeutic timing is central to this framework. Conventional T‐cell‐targeted immunomodulation is most plausible in Stages 1 and 2, when recruitment, effector activation, Th17/Treg imbalance, and DC–T‐cell priming remain active and potentially redirectable. Established Stage 3 fibrosis is more difficult to reverse clinically [3, 4, 181], because human single‐cell and spatial studies show increasing dominance of scar‐forming stromal programs and fibrotic microenvironments [18, 19, 20]. At this stage, T‐cell‐directed strategies are better positioned as progression‐slowing, immune‐resetting, or adjunctive remodeling approaches rather than as stand‐alone scar‐reversal therapies.

### 6.1 Stage 1: Blocking Early Homing and Initial Activation

Engineered extracellular vesicles may further improve tissue‐selective delivery of immunomodulatory or repair cargo to inflamed renal tissue. Current supporting evidence includes Treg‐derived extracellular vesicles that attenuate alloreactive T‐cell responses in transplantation [161], MSC‐derived exosomes that modulate Tregs and the immune microenvironment after AKI [172], and PMSC‐derived extracellular vesicles that attenuate UUO‐associated fibrosis by regulating CD4^+^ T‐cell polarization [173]. Comparative biodistribution and safety data remain needed.

Metabolic intervention may also be useful early. mTOR/AMPK‐directed modulation could restrain activation‐associated glycolysis and effector differentiation, although direct renal T‐cell specificity remains incompletely defined [30, 53]. The glutamine antagonist JHU083 keeps renal T cells in a low‐proliferation state by inhibiting hexokinase II (HK2), CPT1a, and mTOR activity, thereby alleviating ischemic and nephrotoxic AKI. This supports glutamine blockade as a strategy distinct from direct glycolysis inhibition [101]. By contrast, inhibition of PFKFB3 with 3PO suppresses the histone lactylation‐NF‐κB pathway, limits the conversion of excessive glycolysis into chromatin‐linked fibrotic programs, and reduces tubular inflammation and interstitial fibrosis in IRI [105].

### 6.2 Stage 2: Reshaping Subset Balance and Function

In Stage 2, therapy aims to correct T cell polarization before the fibrotic microenvironment becomes self‐sustaining. Dapagliflozin has clinical outcome evidence in CKD [162], and SGK1‐linked Th17/Treg modulation has been shown in diabetic kidney disease models [84]. Orai1 inhibition is supported by preclinical and human lymphocyte data showing reduced IL‐17‐linked kidney injury [66], whereas abatacept has preclinical renal evidence for blocking B7–CD28 costimulation and reducing T cell infiltration [163, 164]. Treg‐supportive approaches include CD28 superagonism after renal IRI [86], low‐dose IL‐2‐based Treg expansion in transplantation, autoimmunity, and inflammatory disease [166], and VDR‐related transplant observational evidence [165]; their relevance to renal fibrosis therefore remains indirect. Selected epigenetic strategies, including HDAC modulation and miPEP31‐linked promotion of Treg differentiation, remain preclinical or indirect [31, 167]. Anti‐ICOS or broader TLS disruption is mechanistically plausible because renal TLS maturation involves T–B/Tfh interactions and IL‐21‐linked fibroblast activation [32, 99].

Targeting the upstream DC–T cell axis is another strategy with preclinical and observational support. The FLT3 inhibitor AC220 depletes pathogenic CD103^+^ DCs, suppresses CD8^+^ T‐cell activation, and attenuates interstitial fibrosis in experimental CKD. The increase in CD141^+^ DCs in human focal segmental glomerulosclerosis biopsy specimens provides translational support, but direct clinical testing is still lacking [79].

Cell‐based regulatory strategies may also be most relevant in Stage 2, but their evidence must be interpreted by cell type and model. In UUO, myeloid‐derived suppressor cells recruited through the CCL5–CCR5 axis suppressed Th1 effector function by expanding Tregs and delayed EMT and extracellular matrix deposition [75]. Conversely, MDSC‐associated ILT4 signaling has been reported to promote renal fibrosis, underscoring context dependence rather than uniform therapeutic benefit [182]. Pharmacologic Treg expansion with KBL409 or anti‐IL‐2 immune complexes reduces renal macrophage accumulation and shifts macrophage polarization toward an anti‐inflammatory state [183, 184]. These approaches aim to restore immune restraint before fibrotic niches become autonomous.

### 6.3 Stage 3: Modulating Exhaustion, Senescence, and Metabolic Failure

In Stage 3, therapeutic strategies focus on exhaustion‐associated checkpoint remodeling, cellular senescence, tissue residency, and metabolic failure. PD‐1/PD‐L1 signaling has direct renal preclinical support [133], while serum soluble PD‐L1 has human CKD biomarker support [134]. Senescence/SASP control is supported by renal preclinical studies [142, 149, 168], whereas NAD^+^ restoration is supported by nicotinamide‐based renal fibrosis models [169]. TRM‐directed approaches have also been tested in renal or glomerular models targeting IL‐15/CD122 and NLRP3‐dependent TRM programs [28, 137].

Among cell‐based approaches, CD19‐targeted CAR T‐cell therapy has emerging clinical support in autoimmune disease, including refractory SLE and lupus nephritis [174, 175], with preclinical renal immune‐injury support in ANCA‐associated AKI [178]. More fibrosis‐directed strategies remain preclinical: PDGFRβ‐targeted CAR T cells reduced fibrosis in CKD mouse models and human kidney organoids [170], FAP‐targeted CAR T cells or FAP inhibition reduced myofibroblast‐associated matrix deposition in UUO and unilateral IRI models [171], and hydrogel‐delivered FAP‐targeted CAR‐M2 macrophages attenuated renal fibrosis while promoting revascularization in preclinical models [185]. Senolytic uPAR CAR T‐cell therapy provides cross‐organ proof of concept [150, 151], but renal translation will require confirmation of target expression, specificity, and long‐term kidney safety. Together, these approaches suggest a possible route toward partial scar remodeling in Stage 3, although clinical reversal of established renal fibrosis has not yet been demonstrated.

### 6.4 Systemic and Multi‐Target Strategies

Some interventions do not map neatly onto a single disease stage because they act on multiple immune and metabolic nodes. Traditional Chinese medicine and natural products may provide promising multi‐target adjunctive strategies for immune and metabolic modulation in renal fibrosis. Qingshen Granules have clinical immune‐function data in selected chronic renal failure/damp‐heat cohorts [90] and renal‐fibrosis mechanistic evidence in adenine‐induced models involving dendritic‐cell metabolism and PI3K/Akt/mTOR signaling [65]. However, formula complexity, population specificity, and standardization limit generalization. Reported natural‐product studies include Cordyceps protein, which reduced T‐cell infiltration and Th1 differentiation in lupus nephritis mice through TLR4/MYD88/MAPK, IL‐12–STAT4, IFN‐γ–STAT1, and PI3K–AKT pathways [179], and Jin‐Gui‐Shen‐Qi‐Wan, which alleviated diabetic kidney fibrosis through mechanisms involving MHC class II [180]. Beyond pharmacologic intervention, dietary sodium restriction may be most relevant where high‐salt/SGK1 biology is active [67], while potassium balance should be managed carefully in CKD [186].

## 7 T‐Cell Biomarkers for Diagnosis and Prognosis

Candidate biomarker readouts are summarized in Table 4. A major translational goal of this framework is to connect mechanism with clinically useful biomarkers for early diagnosis, disease stratification, and treatment‐response monitoring. Because different assays are supported in different renal contexts, Table 4 distinguishes transplant surveillance markers, immune‐mediated or glomerular markers, AKI‐to‐CKD/native‐CKD readouts, and exploratory tissue or peripheral immune signatures.

**TABLE 4:** Candidate T‐cell biomarkers for staging and monitoring renal fibrosis.

Biomarker/readout	Sample/assay	stage signal	Interpretive direction	Evidence and scope	Refs
Granzyme B	Serum; biopsy IHC/RT‐PCR	Cytotoxic T‐cell activity and ECM remodeling	Higher intragraft: cytotoxic allograft injury/ECM remodeling; serum native‐CKD use exploratory.	Transplant strongest; ECM mechanism supportive	[69, 73, 130]
Th17/Treg ratio	Peripheral blood flow cytometry	Stage 2 immune polarization imbalance	Higher ratio: effector skewing; read with IL‐17A/IL‐10 and pathology.	Non‐transplant observational plus renal preclinical	[67, 81, 82, 84]
Banff inflammatory lesions	Renal biopsy Banff i/t/ptc/MVI	Active T‐cell and microvascular injury	Higher i/t: TCMR; ptc/MVI: ABMR‐related microvascular injury.	Transplant‐specific pathology	[74, 187]
Urinary CXCL9/CXCL10	Urine immunoassay; chemokine/creatinine ratio	CXCR3‐axis inflammatory recruitment	Higher urine: allograft inflammation/rejection; native CKD not validated.	Transplant surveillance strongest; renal model support for tissue axis	[131, 188]
IL‐17A and Th17/TLS signal	Serum ELISA; tubular IHC	Stage 2 Th17 activity and TLS amplification	Higher IL‐17A: immune‐active Th17/TLS injury.	Immune‐mediated/glomerular + DKD contexts	[46, 81, 82]
AKI‐to‐CKD inflammatory T‐cell niche	Biopsy or experimental tissue IHC; scRNA‐seq/spatial modules	Persistent inflammation during AKI‐to‐CKD transition	Enriched T‐cell/chemokine modules = unresolved inflammatory recruitment.	Renal preclinical + human/spatial CKD research; not routine assay	[18, 44, 77]
MAIT‐cell abundance/activation	Native kidney biopsy flow cytometry/IHC	Tubulointerstitial immune activation and fibrosis	Higher MAIT activation: fibrosis burden/lower eGFR association.	Human native CKD biopsy evidence	[189]
sPD‐1/sPD‐L1	Serum ELISA	Stage 3 checkpoint shedding and remodeling	Higher sPD‐1/sPD‐L1: checkpoint shedding/remodeling; not protective TEX by default.	IgAN/CKD translational; direction context‐dependent	[97, 133, 134]
TRM density/signature	Biopsy IHC; scRNA‐seq or spatial profiling	Stage 3 tissue residency and reactivation	Pathogenic phenotype matters; density alone is not directional.	Research‐stage FSGS/LN/autoimmune nephritis	[27, 28, 125, 137]
T‐cell senescence/thymic output	Blood flow cytometry; RTE/TREC‐related assays	Stage 3 immune ageing	Low RTEs or high CD4^+^CD28^−^: immune aging/worse CKD prognosis.	Non‐dialysis CKD observational; fibrosis‐specific thresholds untested	[141, 148]
CCL28–Treg repair axis	Tissue/serum profiling; Treg transcriptomics	Immune repair and resolution	Higher repair Treg recruitment: resolution biology after IRI.	Experimental AKI repair/resolution signal	[42]

Note: Biomarker directionality is context‐dependent and should be interpreted with assay type, tissue location, immune phenotype, and longitudinal validation. Abbreviations: ABMR, antibody‐mediated rejection; MAIT, mucosal‐associated invariant T cell; MVI, microvascular inflammation; RTE, recent thymic emigrant; sPD‐1, soluble programmed cell death protein 1; sPD‐L1, soluble programmed death‐ligand 1; TCMR, T cell‐mediated rejection; TEX, exhausted T cell; TREC, T‐cell receptor excision circle; TRM, tissue‐resident memory T cell.

Urinary CXCL9/CXCL10 have the strongest support in transplant surveillance [131, 188], whereas Banff inflammatory lesions remain transplant‐specific biopsy readouts [74, 187]. However, AKI‐to‐CKD and native CKD can be represented by tissue inflammatory T‐cell niches identified in renal fibrosis and post‐injury single‐cell or spatial datasets [18, 44, 77], by MAIT‐cell abundance and activation in human fibrotic CKD biopsies [189], and by peripheral thymic‐output or T‐cell senescence readouts associated with CKD progression [141]. IL‐17A/Th17–Treg readouts remain most useful in immune‐mediated, glomerular, or metabolic CKD contexts [81, 82, 84], whereas soluble checkpoint markers should be separated from tissue TEX: serum sPD‐1/sPD‐L1 elevations generally indicate checkpoint shedding or remodeling linked to fibrosis/progression [133, 134], while tissue TEX enrichment may reflect immune restraint in some transplant settings [10].

Table 5 summarizes commonly used operational markers for the major T‐cell subsets and states discussed in this Review. These markers are compiled from established immunophenotyping guidance [190] and the section‐specific literature cited in each row.

**TABLE 5:** Operational markers for major T‐cell subsets and states discussed in renal fibrosis.

T‐cell subset/state	Common surface markers	Additional defining markers	Refs
Th1	CD4^+^, CXCR3^+^	T‐bet/TBX21, IFN‐γ	[91]
Th2	CD4^+^, CCR4^+^ (variable)	GATA3, IL‐4, IL‐13	[92, 93, 94, 95]
Th17/TRM17	CD4^+^, CCR6^+^; renal TRM17 often CD69^+^	RORC/RORγt, IL‐17A, IL‐23R	[72, 82]
Treg	CD4^+^, CD25^high^, CD127^low^	FOXP3, CTLA‐4, IL‐10	[86]
Tfh/Tph	Tfh: CXCR5^+^, PD‐1^+^, ICOS^+^; Tph: CXCR5^−^, PD‐1^high^	BCL6 or CXCL13	[25, 32]
Cytotoxic CD8^+^/TEM	CD8^+^, CXCR3^+^/CCR5^+^; CD45RO^+^/CCR7^−^ for TEM	GZMB, PRF1, IFNG, TNF	[40, 45, 52, 73]
TRM	CD69^+^, CD103/ITGAE variable, CXCR6^+^, CD38^+^/CD39^+^	CD49a/ITGA1, local retention genes	[27, 28, 125, 137]
TEX	PD‐1^+^, TIGIT^+^, TIM‐3^+^/^−^, LAG‐3^+^, 2B4^+^/CD244^+^	TOX, NR4A, TCF‐1 in progenitors	[10, 121, 122, 123, 126, 127]
Senescent T cells	CD28^−^, CD27^−^, CD57^+^, KLRG1^+^	p16/p21 programs; telomere or DNA‐damage signals	[141, 191]

Note: These markers are practical, non‐exclusive identifiers. Subset assignment should integrate surface phenotype, transcription factors or cytokines, tissue localization, activation state, species, and assay context. Abbreviations: TEM, effector memory T cell; TEX, exhausted T cell; Tfh, T follicular helper cell; Tph, T peripheral helper cell; TRM, tissue‐resident memory T cell; Treg, regulatory T cell.

## 8 Knowledge Gaps and Future Perspectives

Considerable progress has been made in defining the contribution of T cells to renal fibrosis. The field now needs to move beyond whether T cells participate in fibrogenesis and address a more translational question: how these immune programs can be measured, staged, and redirected in patients. The three‐stage model proposed here should therefore be regarded as a mechanistic framework for hypothesis generation, biomarker development, and therapeutic staging rather than as a validated clinical classification.

Several limitations should be acknowledged. First, the framework has not yet been validated longitudinally in human CKD. Human kidney atlases and fibrosis maps now provide important patient‐tissue anchors, but most single‐cell and spatial datasets remain cross‐sectional rather than longitudinal staging cohorts [18, 19, 20]. Second, disease etiology is likely to shape how patients enter or move through the model, because immune‐mediated glomerulonephritis, transplantation, DKD, hypertensive nephrosclerosis, and post‐AKI CKD differ in antigenic stimuli, chemokine programs, stromal niches, and treatment exposure; recent kidney‐immunology syntheses and human kidney profiling studies support this disease‐context view [13, 18]. Third, the reversibility of T‐cell states remains uncertain. Regulatory or progenitor exhausted states may remain redirectable according to broader exhaustion biology [123, 124], whereas terminal exhaustion, senescence, and long‐lived TRM programs may require state‐matched or combination strategies in renal fibrosis [135, 158]. These caveats define the framework as a guide for hypothesis testing, biomarker development, and patient stratification.

Future validation will require longitudinal human cohorts that integrate clinical phenotyping with biopsy histology, single‐cell or single‐nucleus RNA sequencing, spatial transcriptomics or proteomics, TCR sequencing, and blood or urine biomarkers. These approaches also have technical limitations. Tissue dissociation can introduce stress artifacts and under‐sample fragile or rare immune states [192, 193], while spatial methods preserve tissue architecture but still face resolution, segmentation, and deconvolution limits in dense inflammatory niches [194, 195]. Single‐nucleus RNA sequencing can help reduce dissociation‐related loss of rare adult kidney cell states [196], and paired scRNA‐seq/ single‐nucleus RNA sequencing, optimized cold dissociation, multiplexed RNA/protein imaging, cellular indexing of transcriptomes and epitopes by sequencing/T‐cell receptor sequencing integration, and tissue‐section or kidney‐slice validation can further reduce these biases, but they do not eliminate them.

A related challenge is translation across species and models. Rodent AKI‐to‐CKD models provide essential causal and timing information [197], but they compress injury and fibrosis into days to weeks, whereas human CKD usually unfolds over years under recurrent and multifactorial injury. Human and mouse immune systems also differ in T‐cell subset composition, activation programs, chemokine‐receptor usage, and tissue‐niche organization [35, 36]. Future studies should therefore compare conserved functional modules, such as recruitment, effector cytokines, checkpoint remodeling, residency, senescence, and immune‐stromal crosstalk, rather than directly transferring mouse markers or timelines to patients. Human kidney atlas studies define patient‐tissue niches and injury‐associated cell states [19, 29], while fibrosis atlases identify stromal and myofibroblast programs that should anchor T‐cell‐stroma hypotheses [18, 20].

Stronger causal evidence is also needed. Many T‐cell states in kidney disease are currently inferred from surface markers, transcriptional profiles, or spatial proximity to injured tubules, fibroblasts, dendritic cells, and macrophages. These data identify candidate interactions but do not prove that a given T‐cell population drives fibrosis rather than accompanies tissue injury. Functional perturbation, lineage tracing, humanized models, organoid or tissue‐slice co‐cultures, and direct testing of MHC II–dependent or other contact‐dependent T cell–fibroblast circuits will be important for separating pathogenic programs from compensatory or reparative responses.

Clinically, this framework should complement, not replace, established kidney‐disease staging. Current care should continue to use Kidney Disease: Improving Global Outcomes cause‐glomerular filtration rate‐albuminuria staging and disease‐specific pathology systems when appropriate [198]. The proposed model is an immune‐mechanistic overlay: within an existing clinical stage, it asks which T‐cell program is active and whether that program adds prognostic or treatment‐response information. Candidate blood, urine, biopsy, and spatial readouts should be validated against clinically relevant outcomes, including progression rate, relapse, therapeutic response, AKI‐to‐CKD transition, infection or viral reactivation, malignancy‐related risk, and allograft safety when relevant.

Therapeutic development should become equally stage aware. Early recruitment, unresolved immune imbalance, and late immune persistence are unlikely to respond to the same strategy, so future studies should test timed, sequential, or rational combination approaches. Engineered T‐cell and synthetic‐biology strategies remain promising but early in renal fibrosis; their translation will require disease‐specific target validation, kidney‐directed delivery, rigorous safety testing, and evidence that immune engineering improves fibrotic outcomes rather than simply changing immune‐cell abundance. The next phase of the field should therefore build longitudinal human atlases across major CKD etiologies, validate biomarker panels that assign patients to biologically meaningful immune states, and design trials around those states.

## 9 Conclusions

This Review frames renal fibrosis as a dynamic immune‐structural process in which T‐cell functions evolve with disease progression. T cells should not be viewed as a single pathogenic compartment: they participate in recruitment, effector activation, immune imbalance, tissue residency, exhaustion, senescence‐associated remodeling, and late profibrotic persistence. This temporal perspective helps explain why broad T‐cell depletion or nonspecific immunosuppression is unlikely to provide durable antifibrotic benefit across heterogeneous CKD settings.

The three‐stage framework provides a way to organize this complexity and link T‐cell biology to fibrotic progression. Stage 1 connects chemokine‐guided recruitment, cytotoxic injury, and metabolic activation with early fibrotic commitment. Stage 2 captures unresolved inflammation, including Th17/Treg imbalance, APC–T‐cell crosstalk, TLS formation, and maladaptive immune amplification. Stage 3 integrates TEX/TRM persistence, senescence, mitochondrial stress, and established profibrotic signaling. These stages should be understood as overlapping functional states rather than fixed chronological intervals, allowing the framework to accommodate differences among AKI‐to‐CKD transition, immune‐mediated nephritis, transplantation, DKD, and other CKD contexts.

The central translational message is that timing and context are decisive. This may help explain why single‐mediator blockade or nonspecific immunosuppression has often produced limited or context‐dependent benefit in heterogeneous CKD. Future studies should move beyond measuring T‐cell abundance alone and define the functional state, tissue location, and interaction partners of disease‐associated T cells. More effective strategies will require identifying which T‐cell program is active, whether it is harmful or reparative, and whether it remains redirectable. In advanced fibrosis, immune‐targeted therapy will probably need to be combined with approaches that address stromal activation, senescence, metabolic stress, and tissue repair. If validated in longitudinal human cohorts, this framework could help connect immune mechanisms with biomarkers, patient selection, and stage‐matched therapeutic design, moving the field from descriptive immune mapping toward more precise strategies to prevent progressive CKD.

## Author Contributions

**Qianhui Li**: conceptualization, methodology, software, data curation, investigation, validation, formal analysis, supervision, visualization, project administration, Writing – original draft, Writing – review and editing. **Junting Guan**: conceptualization, methodology, software, data curation, investigation, validation, formal analysis, writing – original draft, writing – review and editing. **Yifan Song**: data curation, investigation. **Hongxia Yang**: investigation, data curation. **Guangtao Li**: validation, investigation. **Xiaodong Zhao**: data curation, investigation. **Jiaxin Liu**: visualization. **Bin Liu**: data curation. **Yang Tan**: data curation, visualization. **Honglan Zhou**: resources, project administration, funding acquisition. **Yang‐He Zhang**: funding acquisition, supervision, project administration, resources. **Yishu Wang**: resources, project administration, funding acquisition, supervision.

## Funding

This work was supported by the National Natural Science Foundation of China (Grant Nos. 82470786 and 82270785) and the Postdoctoral Fellowship Program of CPSF (Grant No. GZC20251552).

## Conflicts of Interest

The authors declare no conflicts of interest.

## Data Availability

Data sharing is not applicable to this article because no new data were created or analyzed in this study.
